# Cuproptosis-a potential target for the treatment of osteoporosis

**DOI:** 10.3389/fendo.2023.1135181

**Published:** 2023-05-05

**Authors:** Dinglin Li, Zhonghua Gao, Qian Li, Xiangjie Liu, Hao Liu

**Affiliations:** ^1^ Department of Integrated Traditional Chinese and Western Medicine, Liyuan Hospital, Tongji Medical College, Huazhong University of Science and Technology, Wuhan, China; ^2^ Department of Geriatrics, Liyuan Hospital, Tongji Medical College, Huazhong University of Science and Technology, Wuhan, China

**Keywords:** osteoporosis, copper, cuproptosis, cuproptosis-related genes, inflammation

## Abstract

Osteoporosis is an age-related disease of bone metabolism marked by reduced bone mineral density and impaired bone strength. The disease causes the bones to weaken and break more easily. Osteoclasts participate in bone resorption more than osteoblasts participate in bone formation, disrupting bone homeostasis and leading to osteoporosis. Currently, drug therapy for osteoporosis includes calcium supplements, vitamin D, parathyroid hormone, estrogen, calcitonin, bisphosphates, and other medications. These medications are effective in treating osteoporosis but have side effects. Copper is a necessary trace element in the human body, and studies have shown that it links to the development of osteoporosis. Cuproptosis is a recently proposed new type of cell death. Copper-induced cell death regulates by lipoylated components mediated *via* mitochondrial ferredoxin 1; that is, copper binds directly to the lipoylated components of the tricarboxylic acid cycle, resulting in lipoylated protein accumulation and subsequent loss of iron-sulfur cluster proteins, leading to proteotoxic stress and eventually cell death. Therapeutic options for tumor disorders include targeting the intracellular toxicity of copper and cuproptosis. The hypoxic environment in bone and the metabolic pathway of glycolysis to provide energy in cells can inhibit cuproptosis, which may promote the survival and proliferation of various cells, including osteoblasts, osteoclasts, effector T cells, and macrophages, thereby mediating the osteoporosis process. As a result, our group tried to explain the relationship between the role of cuproptosis and its essential regulatory genes, as well as the pathological mechanism of osteoporosis and its effects on various cells. This study intends to investigate a new treatment approach for the clinical treatment of osteoporosis that is beneficial to the treatment of osteoporosis.

## 1 Introduction

The balance of osteoclast-mediated bone resorption and osteoblast-mediated bone formation is directly responsible for maintaining bone homeostasis (1). Disruptions in this balance caused by over-absorption or under-formation result in decreased bone mass and deterioration of bone tissue microarchitecture, resulting in osteoporosis(OP) (1, 2). Osteoporotic fractures usually threaten people’s health, especially in older individuals, and impose significant socioeconomic burdens (3). Currently, the most common methods for treating OP include inhibiting osteoclast activity and inducing bone formation (4). Inhibitors that osteoclast differentiation and activation, such as estrogen, bisphosphates, denosumab, calcitonin, and others, have been used in the clinic for many years, but they have numerous side effects (4, 5). Drugs that promote bone formation, such as parathyroid hormone and strontium ranelate, also pose many problems in clinical use, such as nausea, dizziness, and leg cramps with long-term use of the parathyroid hormone (6). The manufacturer recalled Strontium ranelate as early as 2017 because long-term use was toxic to the circulatory system (7). As a result, it is advantageous to investigate new drugs and elucidate novel mechanisms of action in the treatment of OP.

Cell death is linked to OP. For example, ferroptosis disrupts osteoblast differentiation (8), and inducing osteoclast apoptosis can maintain bone mass (9). A new type of cell death, known as “cuproptosis,” has recently been proposed (10). In contrast to other recognized death mechanisms, such as pyroptosis and necroptosis, it is a distinct non-apoptotic programmed death mechanism (11). copper initiates it and requires the mediation of protein lipoylation in the mitochondria; specifically, the tricarboxylic acid (TCA) cycle’s lipoylated components are directly bound by the copper to cause cuproptosis, resulting in lipoylated protein aggregation and subsequent loss of iron-sulfur cluster protein, which inevitably leads to proteotoxic stress and eventually cell death (10). In addition, using genome-wide CRIPSR-Cas9 dysfunction screening, Cuproptosis was linked to 10 essential genes, including regulator Ferredoxin 1(FDX1) and six lipoylated protein genes encoding either lipoic acid pathway elements (three key lipoic acid pathway enzymes - Lipoyltransferase 1(LIPT1), Lipoic Acid Synthetase(LiAS), and Dihydrolipoamide Dehydrogenase(DLD) or lipoylated protein targets (three components of the pyruvate dehydrogenase complex, including Dihydrolipoamide S-acetyltransferase (DLAT), Pyruvate Dehydrogenase E1 Subunit Alpha 1(PDHA1), and Pyruvate Dehydrogenase E1 Subunit Beta(PDHB), and knockdown of the above seven genes could rescue cuproptosis, it also includes the negative regulators Metal Regulatory Transcription Factor 1(MTF1), Glutaminase(GLS), and Cyclin-Dependent Kinase Inhibitor 2A(CDKN2A) (10). Furthermore, 728 postmenopausal women(aged between 45 and 80 years old) were divided into two groups based on whether they had OP; the bone mineral density and blood copper content of these women were evaluated, and the results showed that the serum copper level of osteoporotic women was lower than that of healthy women (12). There was a statistical difference between the two groups, and in addition, there was a positive correlation between serum copper level and bone mineral density (12). Similarly, some studies have found that elevated serum copper concentration negatively correlates with the risk of OP (13, 14). Furthermore, some researchers investigated the link between copper intake and OP; the findings revealed that dietary and total copper intake was positively correlated with increased bone mineral density in adults and negatively related to the risk of OP (15). Through the above studies, we found that serum copper levels in patients with OP were low, and total copper intake also affected OP. Therefore, we searched the Web of Science, Pubmed, and other databases for copper, cuproptosis, and cuproptosis-related genes to elucidate the potential link with osteoporosis and explore new therapeutic avenues.

## 2 The potential link between copper and OP

Our bodies contain between 50 and 120 mg of copper, with muscles and bones accounting for roughly two-thirds of the copper content (16, 17). Copper is essential for energy metabolism at the cellular level because its primary function is to constitute the enzyme that transfers electrons (oxidase) to reduce molecular oxygen (18). Among these enzymes, we discovered lysyl oxidase, a copper-dependent monoamine oxidase that can use lysine and hydroxylysine (found in collagen and elastin) as substrates to produce cross-links required for the development of connective tissues such as bones (19–21). In addition, copper is a cofactor for many enzymes in collagen synthesis, especially metalloenzymes (22). Because it constitutes an essential element, its deficiency may lead to bone metabolism disorders and contribute to OP development (23, 24). Several *in vitro* studies have found that copper has a positive effect on the regulation of bone metabolism cells, and copper has been found to stimulate the differentiation of mesenchymal stem cells into the osteogenic lineage (25). Furthermore, copper is a cofactor of antioxidant enzymes that scavenge bone radicals and promote osteoblast activity (26). However, it has been noted that the beneficial effects of copper are dose-dependent, with low doses promoting osteoblast growth and high doses causing cytotoxicity (27). Furthermore, Li et al. discovered that copper deficiency might reduce superoxide dismutase activity in the antioxidant enzyme system, resulting in osteoclast activation and increased bone resorption (18). Therefore, the trace element copper appears to be linked to OP, which intrigued our interest in the concept of “cuproptosis,” recently proposed by Tsvetkov et al. (10).

## 3 The potential link between cuproptosis and OP

**Figure 1:**
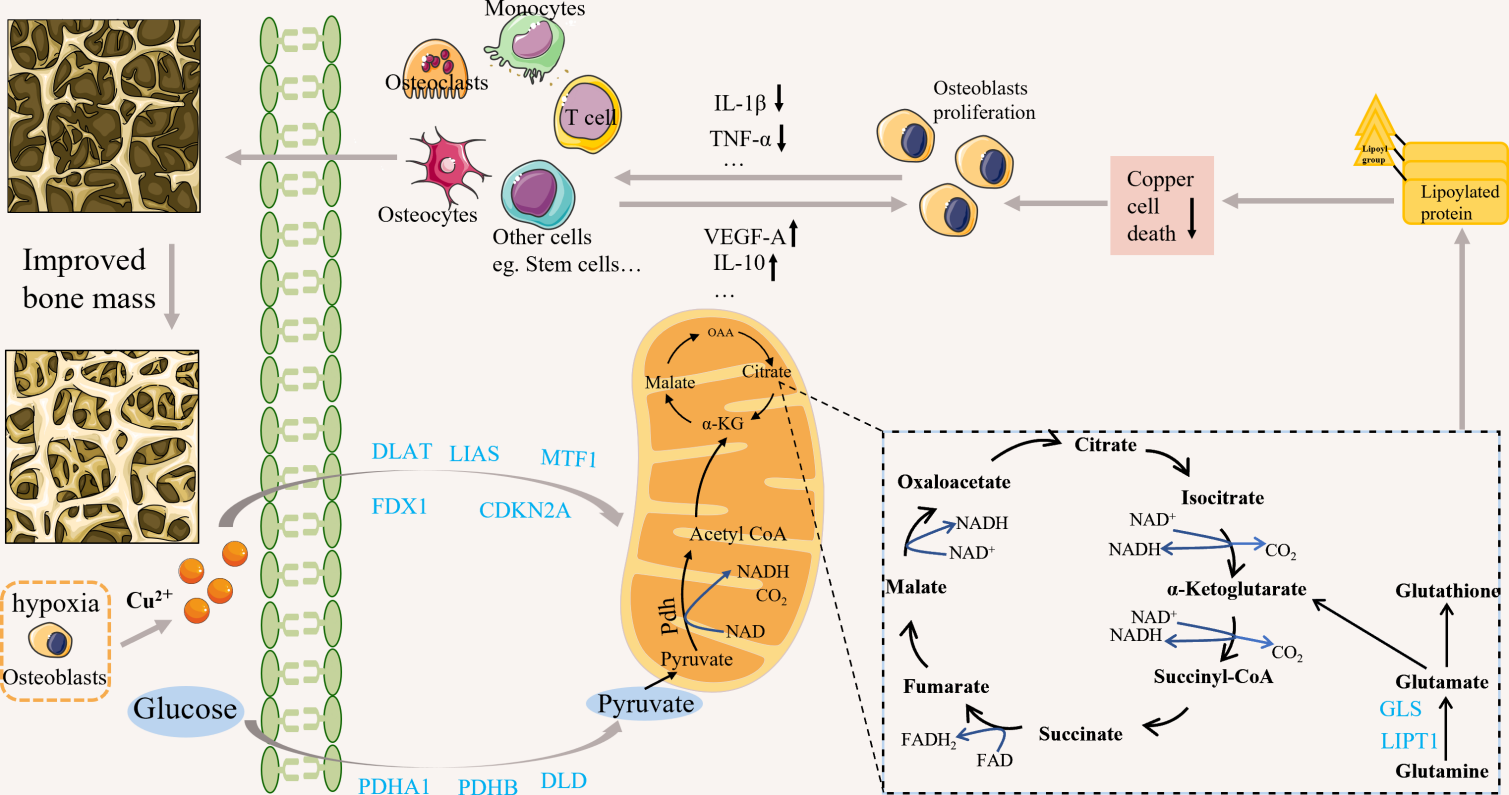
Possible correlation between crucial genes related to cuproptosis and OP. Copper can induce cuproptosis by affecting the TCA cycle. Cuproptosis may be associated with osteoporosis by affecting cell function; for example, it may alleviate osteoporosis by affecting the secretion of cellular inflammatory and angiogenic factors. cuproptosis-related genes may be associated with osteoporosis through various mechanisms, but the specific mechanisms need further study.

Tsvetkov et al. provided a detailed description of cuproptosis (10). First, copper ionophore-induced cell death primarily depends on intracellular copper accumulation (10). Second, copper ionophores cause a distinct type of regulated cell death that differs from other known death mechanisms (10). Furthermore, cells that rely on mitochondrial respiration are nearly 1000-fold more sensitive to copper ionophore-induced cell death than cells that rely on glycolysis; however, mitochondrial respiration can be inhibited under various conditions, including hypoxia, mitochondrial antioxidants, inhibitors of mitochondrial function, and the presence of fatty acids, in addition, glutathione depletion can lead to copper-dependent cell death (10). oligomers’ accumulation is also critical for the development of cuproptosis (10). Finally, the knockdown of 7 genes (including “FDX1, LIPT1, LIAS, and DLD, as well as DLAT, PDHA1, and PDHB”) rescued copper-induced cell death (10). According to research on the epidemiology and pathogenesis of OP, the associated elevated ROS can cause OP (28). Studies have shown that hypoxia is critical in protecting bones from ROS-mediated damage (29), while bones and bone marrow cavities are in natural hypoxia. Recently, it has been shown that osteoblasts and osteoclasts, which maintain bone homeostasis, are oxygen-sensing cells (30), and hypoxia regulates bone remodeling and bone remodeling turnover (31). However, continuous exposure to hypoxic conditions will increase (NF)-kB-ligand(RANKL) formation, promoting osteoclast formation (32). OP occurs due to changes in the bone marrow microenvironment and dysregulation of homeostasis among cells. The metabolic mechanisms associated with cuproptosis may be related to these cells (33). For example, glutamine plays an essential role in the energy metabolism of osteoblasts (34). Biltz RM et al. early found active glutamine absorption and metabolism in explants of the calvaria and long bones (34). Recent studies have shown that matrix mineralization in calvarial osteoblast cultures requires glutamine (35). Stable isotope tracer experiments provide that converting glutamine into citrate thus contributes to the production of energy in the mitochondria of osteoblast precursors (36). Glutamine is also associated with cuproptosis, and a reduction in its level significantly inhibits cuproptosis. Therefore, it may affect the energy metabolism of osteoblasts. Hypoxia may also inhibit cuproptosis (10), but the relationship between hypoxia and copper is very complex. Hypoxia can inhibit the antioxidant defense mechanism by increasing ROS, thus promoting the cytotoxicity of copper (37). Many studies have found that ROS-induced oxidative stress plays a vital role in OP. ROS can indirectly affect osteoclast differentiation, survival, and activation by stimulating bone formation-associated cells to produce osteoprotegerin, macrophage colony-stimulating factor (M-CSF), and RANKL, which are important regulatory factors that recognize osteoclast precursor cells and osteoclasts to conduct bone resorption signals. In addition, effector T cells function through an mTOR-dependent pathway, using glycolytic uptake of glutamine and glucose for energy, which may also inhibit cuproptosis (33). In recent years, some people have studied the relationship between the occurrence and development of OP and T cells. In addition, M1 pro-inflammatory macrophages are glycolytic cells that can inhibit cuproptosis while releasing various pro-inflammatory factors involved in the development and progression of OP. These factors may link OP to cuproptosis (**Table 1**). In addition, a possible correlation between crucial genes related to cuproptosis and OP is described (**Figure 1**).

**Table 1:** The direct or indirect link between cuproptosis-related genes and OP.

Genes related to cuproptosis	The bridge between genes and OP	The relationship between genes and OP
PDHA1	Osteoblasts, osteoclasts, macrophages	PDHA1 inhibition may contribute to osteoblast proliferation; PDHA1 activation affects osteoblasts and macrophages by inhibiting inflammatory factor release.
PDHB	Treg cells, macrophages, osteoblasts	DJ-1 binds to PDHB in Tregs to maintain Treg cell differentiation and T cell integrity. PDHB may affect macrophages by inhibiting ERK signaling; PDHB may affect osteoblast differentiation by affecting mitochondrial homeostasis.
GLS	BMSCs, osteoblasts, osteoclasts, osteocytes, chondrocytes, macrophages	GLS1 affects cells by promoting glutamine metabolism, redox homeostasis, efferocytosis, and antioxidant function.
LIAS	Treg cells, CD4+T cells, macrophages	LIAS is mainly associated with oxidative stress and inflammation.
DLAT	osteoclasts	DLAT may affect OP mainly by influencing the conversion of pyruvate to acetyl-CoA in PDC and mitochondrial metabolism.
FDX1	Macrophages, Treg cells, CD4+T cells	FDX1 may affect cells through metabolic, immune-related pathways.
CDKN2A	Bone marrow adipocytes, BMSCs, osteoblasts, osteoclasts, macrophages, etc.	The P16(INK4A) protein encoded by CDKN2A is a typical marker of cellular senescence- release of senescent cells and inflammatory factors involved in the progression of osteoporosis.
DLD	Osteoclasts, osteoblasts	DLD is associated with osteoporosis through antioxidation and affects energy production in cell metabolism. In addition, DLD can also participate in the treatment of osteoporosis by promoting osseointegration.
MTF1	Osteoblasts, osteoclasts, macrophages, etc.	MTF1 has a direct or indirect relationship with osteoporosis by controlling the balance of metal ions and redox, regulating the expression of related genes, and protecting against metal toxicity.
LIPT1	Osteoblasts, osteoclasts, macrophages, etc.	LIPT1 may be involved in osteoporosis by participating in the tricarboxylic acid cycle and affecting mitochondrial energy metabolism.

### 3.1 The potential link between PDHA1 and OP

PDHA1 may be associated with OP through glycolysis. PDHA1 is a pyruvate dehydrogenase complex(PDC) member, which is essential in glucose metabolism and involves mitochondrial oxidative phosphorylation and the TCA cycle (38). Inhibition of PDHA1 can promote the glycolysis of tumors by down-regulating the activity of PDC (39), increasing glucose and glutamine consumption, and inhibiting oxidative phosphorylation (40). During oxidative phosphorylation, the PDC converts pyruvate to acetyl coenzyme A (33). PDHA1 is a crucial element that connects glycolysis and the TCA cycle (41). Glycolysis converts glucose to pyruvate, which is further converted to acetyl coenzyme A or lactic acid fermentation, depending on aerobic or anaerobic conditions (42). Glucose has long been considered the primary nutrient for osteoblasts; Borle AB et al. showed that bone explants and primary cultured calvaria osteoblasts consume glucose rapidly (43). Zoch ML et al. confirmed the notable glucose absorption by mouse bone (44). Glut transporters are primarily responsible for glucose uptake in osteoblast lineage cells. One study showed that Glut1 and Glut3 were detected in osteoblast cell lines (45). Lactate is the principal end product of glucose metabolism in osteoblasts, regardless of oxygen conditions (46). Historical studies of bone slices or primary calvaria osteoblasts have reported that most glucose carbon is secreted in lactate, even in large amounts of oxygen (47). The production of lactate from glucose under conditions of sufficient oxygen is similar to the Warburg effect observed in many cancer cells, also known as aerobic glycolysis (48). In line with this study, Guntur et al. demonstrated that aerobic glycolysis is the main mode of glucose metabolism in primary calvarial osteoblasts (49). Notably, stimulation of aerobic glycolysis by stabilizing Hifa in preosteoblasts increased osteoblast production and bone formation in mice. Therefore, it can be confirmed that glycolysis is the primary metabolic pathway to meet the needs of ATP during osteoblast differentiation. Transcription factor RUNX family transcription factor 2 (RUNX2) can promote osteoblast differentiation and plays an essential role in bone development and metabolism. In addition, RUNX2 can promote the proliferation of tumor cells by inhibiting the expression of PDHA1 and mitochondrial oxygen consumption rate (50). The glucose metabolism in osteoblasts is similar to that in tumor cells, both of which promote cell proliferation indirectly or directly by enhancing the glycolysis pathway. Therefore, we speculate that PDHA1 may be involved in the proliferation and differentiation of osteoblasts by interfering with the process of glycolysis, and PDHA1 in osteoblasts maybe is suppressed, leading to the glycolysis and proliferation of osteoblasts to affect the course of OP patients directly.

PDHA1 may be associated with OP by regulating inflammation. The NLR Family Pyrin Domain Containing 3(NLRP3) inflammasome was first reported and studied by Martinon and his team in 2002, and it is a multiprotein complex that can respond to physiological and pathogenic stimuli in the body (51). A recent study reported that the NLRP3 inflammasome plays a crucial role in bone resorption (52); and plays a vital role in OP (53, 54). Lin et al. found that when cells were stimulated with NLRP3 inflammasome agonists, lactate fermentation was activated to produce lactate, which induced PKR phosphorylation, activating NLRP3 inflammasomes; in short, activation of NLRP3 inflammasomes requires fermentation of lactic acid (42). Inhibition of PDHA1 will damage pyruvate conversion to acetyl coenzyme A (33). In addition, NLRP3 inflammasomes play a crucial role in host defense by promoting caspase I activation and maturation of IL-1β and IL-18, and dysregulation of their activity leads to auto-inflammatory diseases (55). OP is a chronic inflammatory disease. Macrophages are one of the most critical antigen presenters in the body and are an essential tool for the body to remove pathogenic microorganisms. Osteoclasts are differentiated from mononuclear macrophages, highly differentiated multinucleated giant cells directly involved in bone resorption. Macrophages, the precursor cells of osteoclasts, contain receptor activator of nuclear factor-κB (RANK), which can be induced to form osteoclasts *via* M-CSF and receptor activator of RANKL to participate in bone resorption (2). Scholars now generally agree that macrophages in the local microenvironment are mainly divided into classic (M1) macrophages and atypical (M2) macrophages (56). M1-type macrophages mainly play a pro-inflammatory, antibacterial, and antigen-presenting function, while M2-type macrophages mainly play an inhibitory role in inflammation and tissue repair (57). Recently, Cui et al. found that bionic nanovesicles transforming skeletal endothelial cell-associated secretory phenotypes can treat OP due to suppressed M1 macrophage polarization and enhanced M2 polarization (58). Wei Tong et al. found that deacetylation of macrophage SIRT-3 at lysine 83 can activate PDHA1 and inhibit the activation of NLRP3 inflammasome and the release of IL-1β (59). In conclusion, PDHA1 may be an effective regulator of glycolysis and inflammation and is regulated by distinct transcriptional mechanisms (33).

### 3.2 The potential link between PDHB and OP

PDHB, like PDHA1, belongs to two E1 subtypes of the pyruvate dehydrogenase complex, located mainly in the mitochondria of cells, which catalyze the conversion of glucose-derived pyruvate to acetyl coenzyme A (60). It is the critical component linking glycolysis and TCA cycle metabolic pathways. Mutations or defects in the PDHB gene can lead to various metabolic diseases (61, 62). on the other hand, Acetyl coenzyme A promotes the expression of inflammatory cytokines by regulating histone acetylation (63). Danileviciute et al. identified deglycase DJ-1 (encoded by the PARK7 gene, a critical familial Parkinson’s disease gene) as a pacemaker regulating pyruvate dehydrogenase (PDH) activity in CD4 regulatory T cells; Deglycase DJ-1 binds to PDHB in Tregs and inhibits the phosphorylation of PDHA, thereby promoting PDH activity and oxidative phosphorylation to keep T cells functional integrity and the differentiation of Treg cells (64). An extracellular signal-regulated kinase (ERK) is a critical member of the mitogen-activated protein kinase MAPKs family, which plays an essential role in the development of OP; Wang et al. found that isobavachalcone can prevent OP by inhibiting the ERK activation, NF-kB pathways, and polarization of M1 macrophage (65). Recently, a study on nasopharyngeal carcinoma showed that PDHB could inhibit RasV12-driven ERK signal transduction and cell growth (66). PDHB is located in mitochondria, and the mutual effect between PDHB and NIMA-associated kinase ten may be indispensable to maintaining mitochondrial function; the knockdown of NIMA-associated kinase ten will damage the respiration of mitochondria (67). There are more mitochondria per unit surface area of osteoclasts than almost any other cell, and the energy needed for osteoclast differentiation mainly comes from mitochondrial oxidative metabolism (68). Therefore, PDHB may affect osteoclast differentiation through interaction with NIMA-associated kinase ten.

### 3.3 The potential link between GLS and OP

Glutamine is the most abundant non-essential amino acid in circulation and has a variety of metabolic uses in cells (69). Glutamine metabolism has emerged as a critical regulator of many cellular processes in diverse pathologies (70). It is initiated by GLS, the primary enzyme responsible for glutamine catabolism (70). The activity of GLS is encoded by two protein isoforms, Kidney type Glutaminase A (KGA, or glutaminase 1, encoded by Gls1), and Liver type Glutaminase (LGA, or glutaminase 2, encoded by Gls2) (70). Huang et al. used quantitative PCR (qPCR) analysis to show that bone marrow mesenchymal stromal cells (BMSCs) express Gls1 at much higher levels than Gls2, Gls2 expression is even negligible under both undifferentiated and differentiated conditions, suggesting that Gls1 encodes most of the GLS activity in BMSCs (70). That Gls1 has a regulatory BMSCs proliferation and osteoblast endowment (70). The mechanism may be because inhibition of GLS significantly reduces the content of downstream metabolite α-ketoglutarate (α-KG), and transaminase-dependent α-KG production is critical for the proliferation, specification, and differentiation of BMSCs (70). In addition, it has been found that micRO-RNA-200a-3p is highly expressed in the serum of OP patients; it can inhibit osteogenic differentiation of BMSCs by targeting GLS, thus accelerating the progression of OP (71). Knockdown of micRO-RNA-200a-3p can promote osteogenic differentiation of BMSCs; GLS overexpression similarly reversed the inhibitory effect of micRO-RNA-200a-3p on osteogenic differentiation (71). miR-206 can directly bind to the 3’-UTR region of GLS mRNA, thereby inhibiting GLS expression and glutamine metabolism; rescue experiments to restore GLS lead to recovery of glutamine metabolism and osteogenic differentiation (72). GLS expression and activity are regulated by Wnt/Mtorc1 signaling during osteoblast differentiation (36). MTOR, a protein synthesis master regulator, affects the transcriptional activity of ERR α (73). Glutaminase (GLS) is the target gene of estrogen-related receptor α (ERR α) (73). ERR α and its coactivator PGC-1 α direct GLS, especially in the early stage of osteogenic differentiation, guide mitochondrial glutamine-dependent anaplerosis and make α-KG production into the TCA cycle (73). In conclusion, mTOR affects the ERRα/PGC-1α/Gls signaling pathway to stimulate mitochondrial glutamine anaplerosis, coordinating energy production to help meet increased energy demand from new protein synthesis and promoting osteogenic differentiation of BMSCs (73). GLS inhibition impairs the induction of osteoblast markers (74). In contrast, Huang et al. found that the ERRα/PGC-1β/Gls regulation axis can drive metabolic adaptation that promotes osteoblast differentiation (75). The effect on osteoblasts or osteoclasts may depend on the coactivator of ERR α, namely PGC-1α or PGC-1β. Hypoxia-inducible factor HIF-1α (a key transcription factor in hypoxia signaling) mediates glutathione synthesis through stable stimulation of GLS, allowing cells to maintain redox homeostasis even during baseline, oxidative or nutritional stress to promote adaptive cellular metabolism, which in turn supports the survival of implanted osteoblasts to promote bone regeneration (76). Like this study, Steve et al. found that GLS1-mediated glutamine catabolism supports anabolic processes and redox homeostasis in osteoprogenitors, thereby promoting the proliferation and differentiation of osteoprogenitors into osteoblasts, at the same time, the deletion of GLS1 leads to an osteoporotic phenotype by impairing bone formation (77). The growth and development of longitudinal bones are through endochondral ossification in the growth plate, during which chondrocytes undergo proliferation, hypertrophy, apoptosis, and eventual replacement by osteoblasts (78). Yue et al. obtained abundant low molecular weight peptides of glutamate/glutamine from sea cucumber intestine by enzymatic hydrolysis and administered orally to adolescent mice and found that sea cucumber intestine promoted the Sox9 entry into the chondrogenic gene enhancer region to accelerate the cell cycle through upregulation of glutamine-mediated histone acetylation and ultimately accelerated growth plate chondrocyte proliferation, effectively promoting longitudinal bone growth, in contrast, deletion of GLS1 would inhibit glutamine metabolism in growth plate chondrocytes and interfere with normal chondrocyte function, ultimately leading to stunted long bone growth (79).

Macrophage reprogramming and phenotypic polarization are controlled by glutaminolysis, an essential metabolic factor (80). Some investigations have revealed that α-KG derives from glutaminolysis is necessary for M2 polarization (81–83). Several regulatory mechanisms have been identified for the involvement of glutamine in macrophage activation (84). P-C H et al. found that α-KG produced by glutaminolysis facilitates M2 phenotype formation through epigenetic reprogramming of M2-specific marker genes mediated by histone demethylase Jmjd3 (81). M-N A et al. have shown that UDP-GlcNAc is essential for the polarization of M2 macrophages because it is responsible for the M2 marker proteins glycosylation, and more than one-half of the nitrogen in UDP-GlcNAc comes from glutamine (85). Feng et al. found that GLS1-mediated glutaminolysis promoted the proliferation and adhesion of mouse BMDMs and the infiltration and activation of M2 macrophages, and promoted the secretion of pro-angiogenic cytokines such as VEGF-A (84). The weakening of the ability of macrophages to clear apoptotic cells (i.e., efferocytosis) will enhance the bone resorption capacity of osteoclasts, which is another crucial factor in OP. Merlin et al. revealed that GLS1-mediated glutaminolysis is essential to promote the clearance of apoptotic cells (i.e., efferocytosis) by macrophages during homeostasis in mice (86). The mechanism involves non-classical glutamine metabolism; thus, it plays a massive role in removing dying cells and maintaining tissue homeostasis (86). There is a link between OP and inflammation *in vivo*; in other words, OP is also known as a chronic inflammatory disease. ROS are the product of oxygen metabolism, and high concentrations of ROS will disrupt the balance of oxidants and antioxidants, leading to inflammatory diseases. NF-kB is another critical factor regulating inflammation. Glutamine metabolism is also significant during inflammation, as energy, biosynthesis, and antioxidant capacity are essential for an appropriate immune response (87). Of course, because glutaminase plays an essential role in metabolism and antioxidant function, its transcription is also tightly regulated (88, 89). Merlin et al. found that GLS1 overexpression can limit ROS production (86). In a rat liver transplantation model, DM-αKG (a cell-permeable analog of α-KG) perfusion inhibited NF-kB activity, up-regulated p-GSK3β and Suppressor Of Cytokine Signaling 1(SOCS1) expression in Kupffer cells, shifted M1/M2 balance toward an anti-inflammatory direction, and inhibited serum secretion of pro-inflammatory cytokines and increased IL-10 (90). We also found that some studies have shown that cytokines that promote OP can up-regulate the expression of GLS1. For example, STAT1 is one of the candidate genes for human OP (91). It has been reported that INF-α in monocyte-derived macrophages (MDMs) can increase human GLS1 promoter activity through STAT1 phosphorylation, thereby enhancing GLS1 expression and glutamate production (92, 93). In addition, IL-1β and TNF-α can up-regulate GLS1 expression, which induces glutamate production leading to neurotoxicity (94). In conclusion, GLS1 affects multiple cells mainly through glutamine metabolism and antioxidant function, thus influencing the course of OP.

### 3.4 The potential link between LIAS and OP

LIAS is a Fe-S cluster protein that catalyzes the final step in lipoic acid biosynthesis (95). Lipoic acid(LA) is an essential metabolite in various biochemical actions, such as antioxidants. It performs various cellular functions in various metabolic pathways (95), involving the decarboxylation reactions of pyruvate and α-KG (96, 97). It plays a vital role in mitochondrial energy metabolism (98). Mitochondria are known to be the “energy factory” of the cell, providing energy through oxidative phosphorylation and ATP synthesis. In addition, mitochondria are the primary source of ROS and the most direct target of ROS (99). Oxidative stress occurs when there is an imbalance between the production of ROS and the scavenging capacity of the cellular antioxidant system, and it is thought to be a pathogenic factor in many disease states, including OP (100). LIAS is mainly related to oxidative stress and inflammation. LIAS overexpression decreases inflammatory responses (including reduced expression of pro-inflammatory cytokines/chemokines and inhibition of NF-κB activity) as well as reduces oxidative stress and enhances antioxidant defenses (including increased production of NRF2 and Lias) to potentially protect mitochondrial function in diabetic nephropathy mice (101). Similar to this study, Zhao et al. found that silica significantly increased oxidative stress in wild-type and Lias^H/H^ mice (LIAS overexpression in mice) (102). However, unlike wild-type mice, Lias^H/H^ mice reduced oxidative stress induced by silica through NRF2 signaling; they downregulated pro-inflammatory cytokine expression by inhibiting NF-κB activation, alleviating the severity of significant pathological alterations in the early stages of silica-induced pulmonary fibrosis (102). Overexpression of LIAS in experimental atherosclerotic mice significantly increased the number of Tregs (which can produce anti-inflammatory cytokines such as IL-10); it was accompanied by a decrease in the infiltration of CD4^+^ T cells (which can produce pro-inflammatory cytokines such as INF-γ) to reduce atherosclerosis (103). Corresponding to this study, as early as 2010, Yi et al. found that genetic reduction of LIAS expression increased atherosclerosis in male apolipoprotein E deficient mice, which was associated with a reduction in endogenous antioxidant defense reservoir due to reduced LIAS expression (104). When LIAS is mutated, it stabilizes HIF-1α in a non-hydroxylated form and leads to HIF-1 activation by inhibiting prolyl hydroxylases (PHDs) activity, which may lead to enhanced glycolytic effects in cells (105). In addition, LIAS’s expression level was reported to be negatively correlated with M1 macrophages (106). The above results suggest that LIAS may be included as a new marker in the research field for OP treatment.

### 3.5 The potential link between DLAT and OP

DLAT is the E2 subunit of the PDC in the glucose catabolism pathway, and it plays a critical catalytic role in converting pyruvate to acetyl-CoA (107). E4 transcription factor 1 is a widely expressed transcriptional regulator that plays a crucial role in cell cycle control and proliferation (108); it can interact with OP *via* P53 (109, 110). E4 transcription factor 1 regulates DLAT, and these two ingredients maybe regulate OP together (111). SIRT2 is a kind of deacetylase with a wide range of physiological functions, and Jing et al. found that SIRT2 deficiency prevents age-related bone loss in rats by inhibiting osteoclast production and that DLAT, a substrate of SIRT2, may be involved in regulating the process of OP (112, 113). In addition, component 1Q subcomponent-binding protein in the mitochondria induces activation of the NLRP3 inflammasome (114). It can regulate mitochondrial metabolism by binding to DLAT and affecting the activity of PDH (115). Thus, DLAT may affect OP mainly by influencing the conversion of pyruvate to acetyl-CoA in PDC and mitochondrial metabolism.

### 3.6 The potential link between FDX1 and OP

FDX1, also known as adrenodoxin or hepatoredoxin, is a subunit of the augmin complex and is present in the mitochondrial matrix (116, 117). It encodes a small iron-sulfur protein with low redox potential and molecular weight and contains an iron-sulfur cluster (118). FDX1 transfers electrons from NADPH to mitochondrial cytochrome P450 through the ferredoxin reductase and participates in the metabolism of steroids, vitamin D, and bile acids (119, 120). In addition, FDX1 affects immune cells (e.g., monocytes, M1/M2 macrophages, dendritic cells, neutrophils, mast cells, B cells, regulatory T cells, CD4+ T cells, NK cells, Etc.) and immune-related genes (e.g., CXCL16, CD40, TNFRSF15, 25, CTLA4, CD274, PDCD1, NRP1, Etc.) (116, 117, 121–123). FDX1 was significantly associated with DNA and RNA methylation (e.g., m6A) (121). The FDX1 gene may promote ATP production, and in addition, it is closely associated with the metabolism of glucose, fatty acids, and amino acids (124). FDX1 co-expressed genes are mainly involved in various metabolic processes, such as mitochondrial gene expression and mitochondrial respiratory chain complex assembly, and are associated with the Notch signaling pathway (117). Zhang Zhen et al. used LASSO Cox regression models to construct a novel CRRS (cuproptosis-related risk score) based on FDX1 and its related genes (125). They found that patients in the high CRRS group exhibited cancer-related pathways such as WNT signaling, Notch pathway, and Hedgehog signaling, while many genes encoding glycolytic pathways (e.g., hexokinase 2) were significantly upregulated in hepatocellular carcinoma patients with high CRRS (125). In addition, forkhead box M1, a known transcription factor promoting glycolysis in some cancers by binding to the promoters of glycolytic enzyme genes, including glucose transporters (GLUTs), also increased in patients in the high CRRS group (125). Hypoxia-inducible factor-1 (HIF-1) determines glucose consumption through oxidation or glycolysis, and HIF1A is also elevated in patients in the high-CRRS group (125). In addition, the genes with the highest mutation frequencies in the low CRRS and high CRRS groups included catenin beta one and tumor protein P53 (125). CRRS also showed significant positive correlations with IFNγ markers (IFNG and STAT1), intercellular adhesion molecule 1, and colony-stimulating factor 1 receptor (125). Similar to some of the results of Zhang Zhen et al. is the study by Zhang Chi et al., who found that FDX1 was associated with immune-related pathways such as inflammatory response, IFNγ response, TNF-α/NF-KB signaling pathway (123).

All the genes, cells, or signaling pathways mentioned above are osteoporosis-related. Therefore, FDX1 may be involved in OP through metabolic, immune cells, immune-related genes, and inflammatory pathways.

### 3.7 The potential link between CDKN2A and OP

CDKN2A gene is adjacent to the 9P21.3 genomic region and is a key cell cycle regulator (126). It encodes the P16 (INK4A) protein, a typical marker of cellular senescence (127). The gerontology community increasingly recognizes OP risk factors, including aging (128, 129). Aging reduces the defense against oxidative stress, aggravates the loss of sex steroids, and adversely affects bones (130). Skeletal aging is accompanied by changes in the tissue microenvironment with increased levels of pro-inflammatory cytokines (130), a key factor contributing to OP. Cellular senescence is a basic aging mechanism that can lead to various age-related diseases (131). Cellular senescence has become one of the signs of aging and a major contributor to age-related diseases, including OP (132). Cellular senescence is characterized by growth inhibition, functional alterations, and the presence of so-called senescence-associated secretory phenotypes (SASP), including the expression of inflammatory and trophic factors and tissue remodeling matrix metalloproteinases (MMP) (133). p16 controls the G1 phase of the cell cycle, a key regulator of cellular senescence frequently inactivated in cancer; it is also thought to be associated with many age-related pathologies, including OP (134). The cell cycle-dependent kinase inhibitor p16 is not only a recognized indicator of cellular senescence, but it also acts as a key effector of cellular senescence (127). The expression level of p16 gradually increases during the development of physiological aging and aging-related diseases (135). Senescent cells in the bone microenvironment increase with aging, producing a pro-inflammatory secretion that leads to increased bone resorption and reduced bone formation; methods that eliminate senescent cells or impair their pro-inflammatory secretion production have been shown to prevent age-related bone loss in mice (136). Targeted removal of p16Ink4a-expressing cells in p16-INK-ATTAC (p16Ink4a apoptosis through targeted activation of caspases) mice have been successfully used to understand the effects of removal of p16Ink4a-expressing senescent cells in a variety of age-related diseases, including OP (137, 138). Early observations using the INK-ATTAC model by Farr et al. showed that p16-overexpressing cells in old mice increase the number of osteoclasts (138). p16INK4a directly interacts with and activates the major adipogenic regulator peroxisome proliferator-activated receptor gamma (PPARγ) (139). Eliminating p16-expressing cells in aged INK-ATTAC mice reduces the number of bone marrow adipocytes (138). Similarly, eliminating p16-expressing cells in aged p16-3MR mice (an alternative to the INK-ATTAC model of p16-expressing cell elimination) reduces the adipogenic potential of bone marrow cells (140). The p16-3MR transgenic mouse eliminates senescent osteoclast progenitors but does not eliminate senescent osteocytes (140). However, Farr et al. showed that INK-ATTAC transgenic mice effectively reduced the number of senescent osteocytes in old mice (138). However, it is still unknown whether the elimination of senescent osteocytes themselves is the cause of the increase in bone mass seen in the INK-ATTAC model (140). Yang et al. recently demonstrated that 1,25(OH)_2_D_3_ plays a role in the prevention of age-related osteoporosis by up-regulating Ezh2 through vitamin D receptor-mediated transcription, increasing H3K27me3 and repressing p16 transcription, thus promoting the proliferation and osteogenesis of BMMSCs and inhibiting their senescence, while also stimulating bone formation in osteoblasts and inhibiting osteocyte senescence, SASP, and osteoclastic bone resorption (141). Bmi1 overexpression in MSCs can stimulate MSC proliferation and differentiation into osteoblasts by inactivating p16/p19 signaling and inhibiting oxidative stress, thereby enhancing osteoblast bone formation and exerting anti-osteoporosis effects (142). In addition, c-AbI(a non-receptor tyrosine kinase) regulates BMP-Smad1/5/8 and BMP-Tak1-Mek1/2-Erk1/2 pathways by phosphorylating BMP receptor IA (BMPRIA) to control p16^INK4a^ expression and after that osteoblast expansion and bone formation (143). P16 is the direct target of HIF-2 α in osteoclasts, which mediates the enhancement of bone resorption activity of senescence-related osteoclasts (144). In addition, p16^INK4a^ can regulate the activation and polarization of macrophages (i.e., BMDMs) *via* the JAK2-STAT1 pathway. Its deletion regulates the phenotype of macrophages exhibiting a phenotype similar to IL-4-induced macrophage polarization (i.e., M2 type), resulting in a significant decrease in the expression of inflammatory genes (e.g., IL-6 and TNF) (145). The expression of p16^Ink4a^ is also significantly higher with aging in B cells, T cells, myeloid cells, and osteoblast progenitor cells, leading to bone loss (130). CDKN2A has been linked to OP through the P16 protein it encodes, primarily involved in cellular senescence.

### 3.8 The potential link between DLD and OP

DLD is a member of the redox family of Flavin proteins (146). It can participate in catalysis because the protein consists of 474 amino acids, including an active disulfide bridge and a FAD cofactor, which endow it with activity (147). The normal function of PDC requires the participation of DLD (148). By regulating the components of pyruvate, PDC converts it into acetyl-CoA and guides acetyl-CoA into the Krebs cycle to produce CO2, NADH, and H+; PDC plays an essential role in this process (147). The primary function of DLD is to promote the oxidation of lipoyl molecules by reducing NAD+ to NADH and to prepare for the subsequent cyclic catalytic reaction of lipoyl molecules (147). Therefore, DLD is vital in maintaining cell energy metabolism and the tricarboxylic acid cycle (149). DLD has been proven to have antioxidant properties in humans because it contains important cofactors FAD and NADH (149). In addition, α-lipoic acid is the substrate of DLD, which can treat various chronic diseases, including OP (150, 151). Glucose plays a crucial role in bone metabolism and plays a key role in maintaining the homeostasis between bone formation and bone resorption (152). The enzyme encoded by DLD is part of the PDH enzyme complex, which directly promotes the energy metabolism of glucose (153). Inhibiting the activity of the DLD enzyme decreased the glycolysis catabolism of glucose (153). Glycolysis is an important energy-driven pathway in the process of osteoclasts differentiation (154). Therefore, increasing the process of glycolysis is essential for osteoclasts formation (155), and inhibition of the DLD enzyme may inhibit osteoclasts formation. In addition, it has been found that DLD^RGD^ can be prepared by attaching RGD molecules to the N-terminal and C-terminal of DLD; This conjugate can be coupled with TiO_2_ coated with the implant and promote the osseointegration of osteoblasts and implants through coupling with specific integrins on the surface of bone-forming cells (156–158). Therefore, DLD^RGD^ may be helpful in the treatment of a variety of orthopedic diseases, including osteoporosis. To sum up, DLD is associated with OP through antioxidation and affects energy production in cell metabolism. In addition, DLD can also participate in the treatment of OP by promoting osseointegration.

### 3.9 The potential link between MTF1 and OP

MTF is a zinc finger protein that responds to and regulates various metals, especially zinc, at the cellular level (159–161). As a zinc sensor, MTF1 protects cells from oxidation and hypoxia by inducing the expression of antioxidants and other proteins (162, 163). Therefore, a significant relationship exists between MTF1 and redox metabolism (164). Zinc is needed in many biological processes, such as enzyme activity, gene expression, cell cycle, apoptosis, and immunity (165–167). In addition, zinc can regulate the expression of SOCS3 by activating MTF1 to prevent inflammation (168). MTF1 is the only known transcription factor that mediates zinc-induced gene transcription (169). The content of available zinc in cells is directly related to the existence of metallothioneins (160). Metallothionein is involved in maintaining the balance of metal elements, such as copper, zinc, and cadmium (164); it binds zinc ions and regulates the balance of intracellular zinc ions, affecting many cellular processes, including gene transcription, apoptosis, proliferation, and differentiation (160); it also acts as a redox buffer (164). Metallothionein and metal balance regulation by MTF1 is related to heavy metal exposure (170). Zinc supplementation can activate the binding of MTF1 to DNA and initiate metallothionein mRNA transcription (171). The protein participates in the regular operation of mitochondria by regulating the permeability of the mitochondrial inner membrane (172). In addition, cadmium can be transformed into a non-toxic form by directly binding metallothioneins; metallothioneins can also inhibit cadmium toxicity by reducing cells’ cadmium uptake and counteracting cadmium-induced ROS (173, 174). The accumulation of heavy metals makes MTF1 transfer from the cytosol to the nucleus (175, 176), and MTF1 can bind to the target gene promoter and promote the expression of metallothionein chelators, metal transporters and other metal-induced proteins (177, 178). The content of copper in the cells affects the binding of MTF1 to the target promoter (163). In addition, MTF1 nuclear translocation leads to a decrease in the level of consumable iron to prevent ferroptosis (179). Studies have shown that MTF-1 is the direct target of miR-148-3p, which regulates the transcriptional activity of MTF-1 (180).

In short, metal ions are necessary for the growth and metabolism of bone-related cells. MTF1 has a direct or indirect relationship with osteoporosis by controlling the balance of metal ions and redox, regulating the expression of related genes, and protecting against metal toxicity.

### 3.10 The potential link between LIPT1 and OP

LIPT1 encodes fatty acyltransferase 1 and participates in the lipoic acid metabolic pathway that regulates mitochondrial energy metabolism (181, 182). Among them, it can regulate the transport of LA, an essential substance in mitochondria (181). LA is involved in the TCA cycle, cellular energy metabolism, and glycine decomposition (183). Fatty acyltransferase 1 encoded by LIPT1 can transfer LA to the subunit of the 2-ketoacid dehydrogenase complex (183). If the homolog of LIPT1 disappears, it may lead to the decrease of lipoid of the E2 subunit of the 2-ketoacid dehydrogenase complex (183), and the lack of LIPT1 will inhibit the metabolism of the TCA cycle (184, 185). In addition, LIPT1 supports fat production and balances oxidizing and reducing glutamine metabolism (186).

In short, LIPT1 may be involved in osteoporosis by participating in the tricarboxylic acid cycle and affecting mitochondrial energy metabolism.

## 4 Conclusions and future perspectives

The essential micronutrient copper catalyzes various biological processes. Therefore, almost all cell types require copper for various physiological processes. Tsvetkov et al. recently proposed a new concept of “cuproptosis” and emphasized that it is a regulated form of cell death. Targeted copper toxicity has always been an effective method for treating tumor diseases, and the discovery of cuproptosis will undoubtedly attract more attention. The link between cuproptosis and osteoporosis may include the following. First, cuproptosis may be inhibited in several cells, and this inhibition promotes cell proliferation. Secondly, some critical genes regulating cuproptosis are associated with the developmental process of OP: PDHA1 regulates glycolysis and inflammation; PDHB can inhibit ERK signaling and regulate mitochondrial homeostasis, possibly affecting osteoclast differentiation; GLS mainly affects glutamine metabolism and antioxidant function; LIAS is essential in mitochondrial energy metabolism, mainly related to oxidative stress and inflammation; DLAT may affect OP mainly by influencing the conversion of pyruvate to acetyl-CoA in PDC and mitochondrial metabolism; FDX1 may be involved in osteoporosis through metabolism, immune cells, immune-related genes, and inflammation; CDKN2A is associated with cellular senescence; DLD is associated with osteoporosis through antioxidation, affecting energy production in cell metabolism and promoting bone integration; MTF1 has a direct or indirect relationship with osteoporosis by controlling the balance of metal ions and redox, regulating the expression of related genes, and protecting against metal toxicity; LIPT1 may participate in osteoporosis by participating in the tricarboxylic acid cycle and affecting mitochondrial energy metabolism.

Cuproptosis a recently discovered programmed cell death. Although studies on Cuproptosis have been published one after another, our team has collected and summarized the possible link between cuproptosis and osteoporosis for the first time. However, because the exact mechanism of cuproptosis is unclear, there are no direct reports of cuproptosis and osteoporosis in the clinic. However, there are direct reports of cuproptosis and osteosarcoma (187–189); cuproptosis-related genes and orthopedic-related diseases have been reported, such as rheumatoid arthritis and osteosarcoma (33, 190, 191). Copper levels are up-regulated in various cell types, including bone-related senescent cells and fibroblasts (192). It is one of the characteristics of cellular senescence (192–194); copper sulfate-induced premature senescence (CuSO4-SIPS) always mimics the molecular mechanism of replicative senescence, especially at the endoplasmic reticulum protein homeostasis level (195). Therefore, there is a need for more research to explore the mechanisms underlying cuproptosis that will likely provide new opportunities for clinical application. Regarding therapeutic methods, the development of nanotechnology is exploiting nano-formulations with the cuproptosis mechanism to achieve functional regulation of cells by regulating the intracellular copper content through its delivery capacity. Combining cuproptosis and materials science may inspire a new class of highly effective anti-osteoporosis strategies.

Our team has been committed to studying exosomes, nanovesicles, and nanoparticles’ targeted treatment of osteoporosis. Next, we plan to design a cuproptosis-targeting therapy for osteoporosis. Specific activators or inhibitors of cuproptosis depend on the selected cuproptosis -related genes and cell types. In short, we aim to provide a scientific basis for the clinical development of new strategies for treating osteoporosis; of course, this must be a hopeful and challenging exploration process.

## Author contributions

DL, HL, and XL generated the ideas and wrote the manuscript. ZG and QL participated in the discussions. All authors contributed to the article and approved the submitted version.

## References

[1] Compston JE McClung MR Leslie WD. Osteoporosis. Lancet (London England) (2019) 393(10169):364–76. doi: 10.1016/s0140-6736(18)32112-3 30696576

[2] Boyle WJ Simonet WS Lacey DL. Osteoclast differentiation and activation. Nature (2003) 423(6937):337–42. doi: 10.1038/nature01658 12748652

[3] Taipaleenmäki H Saito H Schröder S Maeda M Mettler R Ring M. Antagonizing microRNA-19a/b augments PTH anabolic action and restores bone mass in osteoporosis in mice. EMBO Mol Med (2022) 14(11):e13617. doi: 10.15252/emmm.202013617 36193848 PMC9641424

[4] Cui Y Guo Y Kong L Shi J Liu P Li R. A bone-targeted engineered exosome platform delivering siRNA to treat osteoporosis. Bioactive materials (2022) 10:207–21. doi: 10.1016/j.bioactmat.2021.09.015 PMC8636739 34901540

[5] Brown C. Osteoporosis: staying strong. Nature (2017) 550(7674):S15–s17. doi: 10.1038/550S15a 28976955

[6] Oryan A Sahvieh S. Effects of bisphosphonates on osteoporosis: focus on zoledronate. Life Sci (2021) 264:118681. doi: 10.1016/j.lfs.2020.118681 33129881

[7] Black DM Rosen CJ. Clinical practice. postmenopausal osteoporosis. New Engl J Med (2016) 374(3):254–62. doi: 10.1056/NEJMcp1513724 26789873

[8] Luo C Xu W Tang X Liu X Cheng Y Wu Y. Canonical wnt signaling works downstream of iron overload to prevent ferroptosis from damaging osteoblast differentiation. Free Radical Biol Med (2022) 188:337–50. doi: 10.1016/j.freeradbiomed.2022.06.236 35752374

[9] Wang L Liu S Zhao Y Liu D Liu Y Chen C. Osteoblast-induced osteoclast apoptosis by fas ligand/FAS pathway is required for maintenance of bone mass. Cell Death differ (2015) 22(10):1654–64. doi: 10.1038/cdd.2015.14 PMC4563780 25744024

[10] Tsvetkov P Coy S Petrova B Dreishpoon M Verma A Abdusamad M. Copper induces cell death by targeting lipoylated TCA cycle proteins. Sci (New York NY) (2022) 375(6586):1254–61. doi: 10.1126/science.abf0529 PMC9273333 35298263

[11] Bao JH Lu WC Duan H Ye YQ Li JB Liao WT. Identification of a novel cuproptosis-related gene signature and integrative analyses in patients with lower-grade gliomas. Front Immunol (2022) 13:933973. doi: 10.3389/fimmu.2022.933973 36045691 PMC9420977

[12] Okyay E Ertugrul C Acar B Sisman AR Onvural B Ozaksoy D. Comparative evaluation of serum levels of main minerals and postmenopausal osteoporosis. Maturitas (2013) 76(4):320–5. doi: 10.1016/j.maturitas.2013.07.015 24011991

[13] Arikan DC Coskun A Ozer A Kilinc M Atalay F Arikan T. Plasma selenium, zinc, copper and lipid levels in postmenopausal Turkish women and their relation with osteoporosis. Biol Trace element Res (2011) 144(1-3):407–17. doi: 10.1007/s12011-011-9109-7 21656042

[14] Gür A Colpan L Nas K Cevik R Saraç J Erdoğan F. The role of trace minerals in the pathogenesis of postmenopausal osteoporosis and a new effect of calcitonin. J Bone miner Metab (2002) 20(1):39–43. doi: 10.1007/s774-002-8445-y 11810415

[15] Fan Y Ni S Zhang H. Associations of copper intake with bone mineral density and osteoporosis in adults: data from the national health and nutrition examination survey. Biol Trace element Res (2022) 200(5):2062–68. doi: 10.1007/s12011-021-02845-5 34283365

[16] Olivares M Uauy R. Limits of metabolic tolerance to copper and biological basis for present recommendations and regulations. Am J Clin Nutr (1996) 63(5):846s–52s. doi: 10.1093/ajcn/63.5.846 8615373

[17] Turnlund JR Keyes WR Peiffer GL Scott KC. Copper absorption, excretion, and retention by young men consuming low dietary copper determined by using the stable isotope 65Cu. Am J Clin Nutr (1998) 67(6):1219–25. doi: 10.1093/ajcn/67.6.1219 9625096

[18] Rondanelli M Faliva MA Infantino V Gasparri C Iannello G Perna S. Copper as dietary supplement for bone metabolism: a review. Nutrients (2021) 13(7):2246. doi: 10.3390/nu13072246 34210051 PMC8308383

[19] Trumbo P Yates AA Schlicker S Poos M. Dietary reference intakes: vitamin a, vitamin K, arsenic, boron, chromium, copper, iodine, iron, manganese, molybdenum, nickel, silicon, vanadium, and zinc. J Am Dietetic Assoc (2001) 101(3):294–301. doi: 10.1016/s0002-8223(01)00078-5 11269606

[20] Dahl SL Rucker RB Niklason LE. Effects of copper and cross-linking on the extracellular matrix of tissue-engineered arteries. Cell Transplant (2005) 14(6):367–74. doi: 10.3727/000000005783982936 16180655

[21] Rucker RB Kosonen T Clegg MS Mitchell AE Rucker BR Uriu-Hare JY. Copper, lysyl oxidase, and extracellular matrix protein cross-linking. Am J Clin Nutr (1998) 67(5 Suppl):996s–1002s. doi: 10.1093/ajcn/67.5.996S 9587142

[22] Pepa GD Brandi ML. Microelements for bone boost: the last but not the least. Clin cases miner Bone Metab (2016) 13(3):181–85. doi: 10.11138/ccmbm/2016.13.3.181 PMC5318168 28228778

[23] Qu X He Z Qiao H Zhai Z Mao Z Yu Z. Serum copper levels are associated with bone mineral density and total fracture. J orthopaedic translation (2018) 14:34–44. doi: 10.1016/j.jot.2018.05.001 PMC6034109 30035031

[24] Mir E Hossein-nezhad A Bahrami A Bekheirnia MR Javadi E Naderi AA. Adequate serum copper concentration could improve bone density, postpone bone loss and protect osteoporosis in women. Iranian J Public Health (2007), 24–9.

[25] Ding H Gao YS Wang Y Hu C Sun Y Zhang C. Dimethyloxaloylglycine increases the bone healing capacity of adipose-derived stem cells by promoting osteogenic differentiation and angiogenic potential. Stem Cells Dev (2014) 23(9):990–1000. doi: 10.1089/scd.2013.0486 24328551 PMC3996975

[26] Zofková I Nemcikova P Matucha P. Trace elements and bone health. Clin Chem Lab Med (2013) 51(8):1555–61. doi: 10.1515/cclm-2012-0868 23509220

[27] Milkovic L Hoppe A Detsch R Boccaccini AR Zarkovic N. Effects of Cu-doped 45S5 bioactive glass on the lipid peroxidation-associated growth of human osteoblast-like cells in vitro. J Biomed materials Res Part A (2014) 102(10):3556–61. doi: 10.1002/jbm.a.35032 24243858

[28] Manolagas SC. From estrogen-centric to aging and oxidative stress: a revised perspective of the pathogenesis of osteoporosis. Endocr Rev (2010) 31(3):266–300. doi: 10.1210/er.2009-0024 20051526 PMC3365845

[29] Yellowley CE Genetos DC. Hypoxia signaling in the skeleton: implications for bone health. Curr osteoporosis Rep (2019) 17(1):26–35. doi: 10.1007/s11914-019-00500-6 PMC6653634 30725321

[30] Arnett TR Gibbons DC Utting JC Orriss IR Hoebertz A Rosendaal M. Hypoxia is a major stimulator of osteoclast formation and bone resorption. J Cell Physiol (2003) 196(1):2–8. doi: 10.1002/jcp.10321 12767036

[31] Stegen S Stockmans I Moermans K Thienpont B Maxwell PH Carmeliet P. Osteocytic oxygen sensing controls bone mass through epigenetic regulation of sclerostin. Nat Commun (2018) 9(1):2557. doi: 10.1038/s41467-018-04679-7 29967369 PMC6028485

[32] Zhu J Tang Y Wu Q Ji YC Kang FW. [Mechanism of participation of osteocytes in the formation of osteoclasts under hypoxia]. Hua xi kou qiang yi xue za zhi = Huaxi kouqiang yixue zazhi = West China J stomatol (2019) 37(5):463–68. doi: 10.7518/hxkq.2019.05.002 PMC7030417 31721490

[33] Zhao J Guo S Schrodi SJ He D. Cuproptosis and cuproptosis-related genes in rheumatoid arthritis: implication, prospects, and perspectives. Front Immunol (2022) 13:930278. doi: 10.3389/fimmu.2022.930278 35990673 PMC9386151

[34] Biltz RM Letteri JM Pellegrino ED Palekar A Pinkus LM. Glutamine metabolism in bone. Miner electrolyte Metab (1983) 9(3):125–31. 6135980

[35] Brown PM Hutchison JD Crockett JC. Absence of glutamine supplementation prevents differentiation of murine calvarial osteoblasts to a mineralizing phenotype. Calcif Tissue Int (2011) 89(6):472–82. doi: 10.1007/s00223-011-9537-6 21972050

[36] Karner CM Esen E Okunade AL Patterson BW Long F. Increased glutamine catabolism mediates bone anabolism in response to WNT signaling. J Clin Invest (2015) 125(2):551–62. doi: 10.1172/jci78470 PMC4319407 25562323

[37] Pan Y Ai CX Zeng L Liu C Li WC. Modulation of copper-induced antioxidant defense, Cu transport, and mitophagy by hypoxia in the large yellow croaker (Larimichthys crocea). Fish Physiol Biochem (2020) 46(3):997–1010. doi: 10.1007/s10695-020-00765-0 31925663

[38] Patel MS Nemeria NS Furey W Jordan F. The pyruvate dehydrogenase complexes: structure-based function and regulation. J Biol Chem (2014) 289(24):16615–23. doi: 10.1074/jbc.R114.563148 PMC4059105 24798336

[39] Yu L Chen X Sun X Wang L Chen S. The glycolytic switch in tumors: how many players are involved? J Cancer (2017) 8(17):3430–40. doi: 10.7150/jca.21125 PMC5687156 29151926

[40] Liu L Cao J Zhao J Li X Suo Z Li H. PDHA1 gene knockout in human esophageal squamous cancer cells resulted in greater warburg effect and aggressive features. In Vitro And In Vivo OncoTargets Ther (2019) 12:9899–913. doi: 10.2147/ott.S226851 PMC6874154 31819487

[41] Liu Z Yu M Fei B Fang X Ma T Wang D. miR−21−5p targets PDHA1 to regulate glycolysis and cancer progression in gastric cancer. Oncol Rep (2018) 40(5):2955–63. doi: 10.3892/or.2018.6695 30226598

[42] Lin HC Chen YJ Wei YH Lin HA Chen CC Liu TF. Lactic acid fermentation is required for NLRP3 inflammasome activation. Front Immunol (2021) 12:630380. doi: 10.3389/fimmu.2021.630380 33854503 PMC8039150

[43] Borle AB Nichols N Nichols G Jr. Metabolic studies of bone *in vitro.* i. normal bone. J Biol Chem (1960) 235:1206–10. 13802861

[44] Zoch ML Abou DS Clemens TL Thorek DL Riddle RC. *In vivo* Radiometric analysis of glucose uptake and distribution in mouse bone. Bone Res (2016) 4:16004. doi: 10.1038/boneres.2016.4 27088042 PMC4820746

[45] Zoidis E Ghirlanda-Keller C Schmid C. Stimulation of glucose transport in osteoblastic cells by parathyroid hormone and insulin-like growth factor I. Mol Cell Biochem (2011) 348(1-2):33–42. doi: 10.1007/s11010-010-0634-z 21076856

[46] Esen E Long F. Aerobic glycolysis in osteoblasts. Curr osteoporosis Rep (2014) 12(4):433–8. doi: 10.1007/s11914-014-0235-y PMC4216598 25200872

[47] Cohn DV Forscher BK. Aerobic metabolism of glucose by bone. J Biol Chem (1962) 237:615–8. doi: 10.1016/S0021-9258(18)60342-4 13880345

[48] Warburg O. On the origin of cancer cells. Sci (New York NY) (1956) 123(3191):309–14. doi: 10.1126/science.123.3191.309 13298683

[49] Guntur AR Le PT Farber CR Rosen CJ. Bioenergetics during calvarial osteoblast differentiation reflect strain differences in bone mass. Endocrinology (2014) 155(5):1589–95. doi: 10.1210/en.2013-1974 PMC3990840 24437492

[50] Choe M Brusgard JL Chumsri S Bhandary L Zhao XF Lu S. The RUNX2 transcription factor negatively regulates SIRT6 expression to alter glucose metabolism in breast cancer cells. J Cell Biochem (2015) 116(10):2210–26. doi: 10.1002/jcb.25171 PMC4885214 25808624

[51] Schlüter A Heuer H Szczepanowski R Forney LJ Thomas CM Pühler A. The 64 508 bp IncP-1beta antibiotic multiresistance plasmid pB10 isolated from a waste-water treatment plant provides evidence for recombination between members of different branches of the IncP-1beta group. Microbiol (Reading England) (2003) 149(Pt 11):3139–53. doi: 10.1099/mic.0.26570-0 14600226

[52] Alippe Y Wang C Ricci B Xiao J Qu C Zou W. Bone matrix components activate the NLRP3 inflammasome and promote osteoclast differentiation. Sci Rep (2017) 7(1):6630. doi: 10.1038/s41598-017-07014-0 28747793 PMC5529467

[53] Li H Huang C Zhu J Gao K Fang J Li H. Lutein suppresses oxidative stress and inflammation by Nrf2 activation in an osteoporosis rat model. Med Sci monitor (2018) 24:5071–75. doi: 10.12659/msm.908699 PMC6067024 30030965

[54] An Y Zhang H Wang C Jiao F Xu H Wang X. Activation of ROS/MAPKs/NF-κB/NLRP3 and inhibition of efferocytosis in osteoclast-mediated diabetic osteoporosis. FASEB J (2019) 33(11):12515–27. doi: 10.1096/fj.201802805RR PMC6902677 31461386

[55] Snouwaert JN Nguyen M Repenning PW Dye R Livingston EW Kovarova M. An NLRP3 mutation causes arthropathy and osteoporosis in humanized mice. Cell Rep (2016) 17(11):3077–88. doi: 10.1016/j.celrep.2016.11.052 27974218

[56] Martinez FO Gordon S. The M1 and M2 paradigm of macrophage activation: time for reassessment. F1000prime Rep (2014) 6:13. doi: 10.12703/p6-13 24669294 PMC3944738

[57] Das A Sinha M Datta S Abas M Chaffee S Sen CK. Monocyte and macrophage plasticity in tissue repair and regeneration. Am J Pathol (2015) 185(10):2596–606. doi: 10.1016/j.ajpath.2015.06.001 PMC4607753 26118749

[58] Cui Y Li Z Guo Y Qi X Yang Y Jia X. Bioinspired nanovesicles convert the skeletal endothelium-associated secretory phenotype to treat osteoporosis. ACS nano (2022) 16(7):11076–91. doi: 10.1021/acsnano.2c03781 35801837

[59] Wei T Gao J Huang C Song B Sun M Shen W. SIRT3 (Sirtuin-3) prevents ang II (Angiotensin II)-induced macrophage metabolic switch improving perivascular adipose tissue function. Arteriosclerosis thrombosis Vasc Biol (2021) 41(2):714–30. doi: 10.1161/atvbaha.120.315337 33327751

[60] Zhu Y Wu G Yan W Zhan H Sun P. miR-146b-5p regulates cell growth, invasion, and metabolism by targeting PDHB in colorectal cancer. Am J Cancer Res (2017) 7(5):1136–50. PMC5446479 28560062

[61] Dung VM Suong DNA Okamaoto Y Hiramatsu Y Thao DTP Yoshida H. Neuron-specific knockdown of drosophila PDHB induces reduction of lifespan, deficient locomotive ability, abnormal morphology of motor neuron terminals and photoreceptor axon targeting. Exp Cell Res (2018) 366(2):92–102. doi: 10.1016/j.yexcr.2018.02.035 29501567

[62] Kikuchi D Minamishima YA Nakayama K. Prolyl-hydroxylase PHD3 interacts with pyruvate dehydrogenase (PDH)-E1β and regulates the cellular PDH activity. Biochem Biophys Res Commun (2014) 451(2):288–94. doi: 10.1016/j.bbrc.2014.07.114 25088999

[63] Langston PK Nambu A Jung J Shibata M Aksoylar HI Lei J. Glycerol phosphate shuttle enzyme GPD2 regulates macrophage inflammatory responses. Nat Immunol (2019) 20(9):1186–95. doi: 10.1038/s41590-019-0453-7 PMC6707851 31384058

[64] Danileviciute E Zeng N Capelle CM Paczia N Gillespie MA Kurniawan H. PARK7/DJ-1 promotes pyruvate dehydrogenase activity and maintains t(reg) homeostasis during ageing. Nat Metab (2022) 4(5):589–607. doi: 10.1038/s42255-022-00576-y 35618940

[65] Wang X Ji Q Hu W Zhang Z Hu F Cao S. Isobavachalcone prevents osteoporosis by suppressing activation of ERK and NF-κB pathways and M1 polarization of macrophages. Int Immunopharmacol (2021) 94:107370. doi: 10.1016/j.intimp.2021.107370 33640858

[66] Tang H Luo X Li J Zhou Y Li Y Song L. Pyruvate dehydrogenase b promoted the growth and migration of the nasopharyngeal carcinoma cells. Tumour Biol (2016) 37(8):10563–9. doi: 10.1007/s13277-016-4922-4 26857147

[67] Peres de Oliveira A Basei FL Slepicka PF de Castro Ferezin C Melo-Hanchuk TD de Souza EE. NEK10 interactome and depletion reveal new roles in mitochondria. Proteome Sci (2020) 18:4. doi: 10.1186/s12953-020-00160-w 32368190 PMC7189645

[68] Lemma S Sboarina M Porporato PE Zini N Sonveaux P Di Pompo G. Energy metabolism in osteoclast formation and activity. Int J Biochem Cell Biol (2016) 79:168–80. doi: 10.1016/j.biocel.2016.08.034 27590854

[69] Stein WH Moore S. The free amino acids of human blood plasma. J Biol Chem (1954) 211(2):915–26. doi: 10.1016/S0021-9258(18)71179-4 13221597

[70] Yu Y Newman H Shen L Sharma D Hu G Mirando AJ. Glutamine metabolism regulates proliferation and lineage allocation in skeletal stem cells. Cell Metab (2019) 29(4):966–78.e4. doi: 10.1016/j.cmet.2019.01.016 30773468 PMC7062112

[71] Lv R Pan X Song L Sun Q Guo C Zou S. MicroRNA-200a-3p accelerates the progression of osteoporosis by targeting glutaminase to inhibit osteogenic differentiation of bone marrow mesenchymal stem cells. Biomed pharmacother = Biomed pharmacother (2019) 116:108960. doi: 10.1016/j.biopha.2019.108960 31112871

[72] Chen Y Yang YR Fan XL Lin P Yang H Chen XZ. miR-206 inhibits osteogenic differentiation of bone marrow mesenchymal stem cells by targetting glutaminase. Biosci Rep (2019) 39(3):bsr20181108. doi: 10.1042/bsr20181108 30804229 PMC6900431

[73] Huang T Liu R Fu X Yao D Yang M Liu Q. Aging reduces an ERRalpha-directed mitochondrial glutaminase expression suppressing glutamine anaplerosis and osteogenic differentiation of mesenchymal stem cells. Stem Cells (Dayton Ohio) (2017) 35(2):411–24. doi: 10.1002/stem.2470 27501743

[74] Chiu M Toscani D Marchica V Taurino G Costa F Bianchi MG. Myeloma cells deplete bone marrow glutamine and inhibit osteoblast differentiation limiting asparagine availability. Cancers (2020) 12(11):3267. doi: 10.3390/cancers12113267 33167336 PMC7694402

[75] Huang T Fu X Wang N Yang M Zhang M Wang B. Andrographolide prevents bone loss via targeting estrogen-related receptor-α-regulated metabolic adaption of osteoclastogenesis. Br J Pharmacol (2021) 178(21):4352–67. doi: 10.1111/bph.15614 34233019

[76] Stegen S van Gastel N Eelen G Ghesquière B D’Anna F Thienpont B. HIF-1α promotes glutamine-mediated redox homeostasis and glycogen-dependent bioenergetics to support postimplantation bone cell survival. Cell Metab (2016) 23(2):265–79. doi: 10.1016/j.cmet.2016.01.002 PMC7611069 26863487

[77] Stegen S Devignes CS Torrekens S Van Looveren R Carmeliet P Carmeliet G. Glutamine metabolism in osteoprogenitors is required for bone mass accrual and PTH-induced bone anabolism in Male mice. J Bone miner Res (2021) 36(3):604–16. doi: 10.1002/jbmr.4219 33253422

[78] Mackie EJ Tatarczuch L Mirams M. The skeleton: a multi-functional complex organ: the growth plate chondrocyte and endochondral ossification. J Endocrinol (2011) 211(2):109–21. doi: 10.1530/joe-11-0048 21642379

[79] Yue H Tian Y Feng X Bo Y Leng Z Dong P. Novel peptides from sea cucumber intestinal hydrolysates promote longitudinal bone growth in adolescent mice through accelerating cell cycle progress by regulating glutamine metabolism. Food Funct (2022) 13(14):7730–39. doi: 10.1039/d2fo01063a 35762389

[80] Ren W Xia Y Chen S Wu G Bazer FW Zhou B. Glutamine metabolism in macrophages: a novel target for Obesity/Type 2 diabetes. Adv Nutr (Bethesda Md) (2019) 10(2):321–30. doi: 10.1093/advances/nmy084 PMC6416106 30753258

[81] Liu PS Wang H Li X Chao T Teav T Christen S. α-ketoglutarate orchestrates macrophage activation through metabolic and epigenetic reprogramming. Nat Immunol (2017) 18(9):985–94. doi: 10.1038/ni.3796 28714978

[82] Liu S Yang J Wu Z. The regulatory role of α-ketoglutarate metabolism in macrophages. Mediators Inflammation (2021) 2021:5577577. doi: 10.1155/2021/5577577 PMC8024083 33859536

[83] Liu M Chen Y Wang S Zhou H Feng D Wei J. α-ketoglutarate modulates macrophage polarization through regulation of PPARγ transcription and mTORC1/p70S6K pathway to ameliorate ALI/ARDS. Shock (Augusta Ga) (2020) 53(1):103–13. doi: 10.1097/shk.0000000000001333 31841452

[84] Feng Y Yang X Huang J Shen M Wang L Chen X. Pharmacological inhibition of glutaminase 1 attenuates alkali-induced corneal neovascularization by modulating macrophages. Oxid Med Cell Longevity (2022) 2022:1106313. doi: 10.1155/2022/1106313 PMC8957416 35345831

[85] Jha AK Huang SC Sergushichev A Lampropoulou V Ivanova Y Loginicheva E. Network integration of parallel metabolic and transcriptional data reveals metabolic modules that regulate macrophage polarization. Immunity (2015) 42(3):419–30. doi: 10.1016/j.immuni.2015.02.005 25786174

[86] Merlin J Ivanov S Dumont A Sergushichev A Gall J Stunault M. Non-canonical glutamine transamination sustains efferocytosis by coupling redox buffering to oxidative phosphorylation. Nat Metab (2021) 3(10):1313–26. doi: 10.1038/s42255-021-00471-y PMC7611882 34650273

[87] Wu B Liu J Zhao R Li Y Peer J Braun AL. Glutaminase 1 regulates the release of extracellular vesicles during neuroinflammation through key metabolic intermediate alpha-ketoglutarate. J Neuroinflamm (2018) 15(1):79. doi: 10.1186/s12974-018-1120-x PMC5853116 29540215

[88] Erickson JW Cerione RA. Glutaminase: a hot spot for regulation of cancer cell metabolism? Oncotarget (2010) 1(8):734–40. doi: 10.18632/oncotarget.208 PMC3018840 21234284

[89] Márquez J de la Oliva AR Matés JM Segura JA Alonso FJ. Glutaminase: a multifaceted protein not only involved in generating glutamate. Neurochem Int (2006) 48(6-7):465–71. doi: 10.1016/j.neuint.2005.10.015 16516349

[90] Cheng MX Cao D Chen Y Li JZ Tu B Gong JP. α-ketoglutarate attenuates ischemia-reperfusion injury of liver graft in rats. Biomed pharmacother = Biomed pharmacother (2019) 111:1141–46. doi: 10.1016/j.biopha.2018.12.149 30841427

[91] Johnson ML Lara N Kamel MA. How genomics has informed our understanding of the pathogenesis of osteoporosis. Genome Med (2009) 1(9):84. doi: 10.1186/gm84 19735586 PMC2768991

[92] Zhao L Huang Y Zheng J. STAT1 regulates human glutaminase 1 promoter activity through multiple binding sites in HIV-1 infected macrophages. PloS One (2013) 8(9):e76581. doi: 10.1371/journal.pone.0076581 24086752 PMC3782442

[93] Zhao L Huang Y Tian C Taylor L Curthoys N Wang Y. Interferon-α regulates glutaminase 1 promoter through STAT1 phosphorylation: relevance to HIV-1 associated neurocognitive disorders. PloS One (2012) 7(3):e32995. doi: 10.1371/journal.pone.0032995 22479354 PMC3316554

[94] Ye L Huang Y Zhao L Li Y Sun L Zhou Y. IL-1β and TNF-α induce neurotoxicity through glutamate production: a potential role for neuronal glutaminase. J neurochem (2013) 125(6):897–908. doi: 10.1111/jnc.12263 23578284 PMC3747774

[95] Hendricks AL Wachnowsky C Fries B Fidai I Cowan JA. Characterization and reconstitution of human lipoyl synthase (LIAS) supports ISCA2 and ISCU as primary cluster donors and an ordered mechanism of cluster assembly. Int J Mol Sci (2021) 22(4):1598. doi: 10.3390/ijms22041598 33562493 PMC7915201

[96] Packer L Witt EH Tritschler HJ. Alpha-lipoic acid as a biological antioxidant. Free Radical Biol Med (1995) 19(2):227–50. doi: 10.1016/0891-5849(95)00017-r 7649494

[97] Reed LJ Debusk BG. Chemical nature of an alpha-lipoic acid conjugate required for oxidation of pyruvate and alpha-ketoglutarate by an escherichia coli mutant. J Biol Chem (1952) 199(2):881–8. doi: 10.1016/S0021-9258(18)38527-2 13022696

[98] Shay KP Moreau RF Smith EJ Smith AR Hagen TM. Alpha-lipoic acid as a dietary supplement: molecular mechanisms and therapeutic potential. Biochim Biophys Acta (2009) 1790(10):1149–60. doi: 10.1016/j.bbagen.2009.07.026 PMC2756298 19664690

[99] Lyu L Bi Y Li S Xue H Li Y Prusky DB. Sodium silicate prime defense responses in harvested muskmelon by regulating mitochondrial energy metabolism and reactive oxygen species production. Food Chem (2019) 289:369–76. doi: 10.1016/j.foodchem.2019.03.058 30955625

[100] Kimball JS Johnson JP Carlson DA. Oxidative stress and osteoporosis. J Bone Joint Surg Am volume (2021) 103(15):1451–61. doi: 10.2106/jbjs.20.00989 34014853

[101] Zhao Y Yan T Xiong C Chang M Gao Q Yao S. Overexpression of lipoic acid synthase gene alleviates diabetic nephropathy of lepr(db/db) mice. BMJ Open Diabetes Res Care (2021) 9(1):e002260. doi: 10.1136/bmjdrc-2021-002260 PMC8240563 34183321

[102] Zhao Y Xu G Li H Chang M Guan Y Li Y. Overexpression of endogenous lipoic acid synthase attenuates pulmonary fibrosis induced by crystalline silica in mice. Toxicol Lett (2020) 323:57–66. doi: 10.1016/j.toxlet.2020.01.023 32017981

[103] Tian S Nakamura J Hiller S Simington S Holley DW Mota R. New insights into immunomodulation via overexpressing lipoic acid synthase as a therapeutic potential to reduce atherosclerosis. Vasc Pharmacol (2020) 133-134:106777. doi: 10.1016/j.vph.2020.106777 PMC8020442 32750408

[104] Yi X Xu L Kim K Kim HS Maeda N. Genetic reduction of lipoic acid synthase expression modestly increases atherosclerosis in male, but not in female, apolipoprotein e-deficient mice. Atherosclerosis (2010) 211(2):424–30. doi: 10.1016/j.atherosclerosis.2010.03.009 PMC2914155 20347443

[105] Burr SP Costa AS Grice GL Timms RT Lobb IT Freisinger P. Mitochondrial protein lipoylation and the 2-oxoglutarate dehydrogenase complex controls HIF1α stability in aerobic conditions. Cell Metab (2016) 24(5):740–52. doi: 10.1016/j.cmet.2016.09.015 PMC5106373 27923773

[106] Cai Y He Q Liu W Liang Q Peng B Li J. Comprehensive analysis of the potential cuproptosis-related biomarker LIAS that regulates prognosis and immunotherapy of pan-cancers. Front Oncol (2022) 12:952129. doi: 10.3389/fonc.2022.952129 35982953 PMC9379260

[107] Goh WQ Ow GS Kuznetsov VA Chong S Lim YP. DLAT subunit of the pyruvate dehydrogenase complex is upregulated in gastric cancer-implications in cancer therapy. Am J Trans Res (2015) 7(6):1140–51. PMC4532746 26279757

[108] Moison C Chagraoui J Caron MC Gagné JP Coulombe Y Poirier GG. Zinc finger protein E4F1 cooperates with PARP-1 and BRG1 to promote DNA double-strand break repair. Proc Natl Acad Sci United States America (2021) 118(11):e2019408118. doi: 10.1073/pnas.2019408118 PMC7980444 33692124

[109] Lacroix M Linares LK Rueda-Rincon N Bloch K Di Michele M De Blasio C. The multifunctional protein E4F1 links P53 to lipid metabolism in adipocytes. Nat Commun (2021) 12(1):7037. doi: 10.1038/s41467-021-27307-3 34857760 PMC8639890

[110] Wang X Kua HY Hu Y Guo K Zeng Q Wu Q. p53 functions as a negative regulator of osteoblastogenesis, osteoblast-dependent osteoclastogenesis, and bone remodeling. J Cell Biol (2006) 172(1):115–25. doi: 10.1083/jcb.200507106 PMC2063539 16380437

[111] Lacroix M Rodier G Kirsh O Houles T Delpech H Seyran B. E4F1 controls a transcriptional program essential for pyruvate dehydrogenase activity. Proc Natl Acad Sci United States America (2016) 113(39):10998–1003. doi: 10.1073/pnas.1602754113 PMC5047171 27621446

[112] Jing Y Zhou Y Zhou F Wang X Tao B Sun L. SIRT2 deficiency prevents age-related bone loss in rats by inhibiting osteoclastogenesis. Cell Mol Biol (Noisy-le-Grand France) (2019) 65(7):66–71. doi: 10.14715/cmb/2019.65.7.12 31880520

[113] Dai Q Zheng Z Xia F Liu P Li M. A one-step specific assay for continuous detection of sirtuin 2 activity. Acta Pharm Sin B (2019) 9(6):1183–92. doi: 10.1016/j.apsb.2019.05.007 PMC6900550 31867164

[114] Fu RH Tsai CW Chiu SC Liu SP Chiang YT Kuo YH. C9-ALS-Associated proline-arginine dipeptide repeat protein induces activation of NLRP3 inflammasome of HMC3 microglia cells by binding of complement component 1 q subcomponent-binding protein (C1QBP), and syringin prevents this effect. Cells (2022) 11(19):3128. doi: 10.3390/cells11193128 36231090 PMC9563448

[115] Chen R Xiao M Gao H Chen Y Li Y Liu Y. Identification of a novel mitochondrial interacting protein of C1QBP using subcellular fractionation coupled with CoIP-MS. Anal bioanal Chem (2016) 408(6):1557–64. doi: 10.1007/s00216-015-9228-7 26753982

[116] Xiao C Yang L Jin L Lin W Zhang F Huang S. Prognostic and immunological role of cuproptosis-related protein FDX1 in pan-cancer. Front Genet (2022) 13:962028. doi: 10.3389/fgene.2022.962028 36061184 PMC9437317

[117] Huang X Wang T Ye J Feng H Zhang X Ma X. FDX1 expression predicts favourable prognosis in clear cell renal cell carcinoma identified by bioinformatics and tissue microarray analysis. Front Genet (2022) 13:994741. doi: 10.3389/fgene.2022.994741 36186457 PMC9523472

[118] Ewen KM Hannemann F Iametti S Morleo A Bernhardt R. Functional characterization of Fdx1: evidence for an evolutionary relationship between P450-type and ISC-type ferredoxins. J Mol Biol (2011) 413(5):940–51. doi: 10.1016/j.jmb.2011.09.010 21945528

[119] Mateo J Steuten L Aftimos P André F Davies M Garralda E. Delivering precision oncology to patients with cancer. Nat Med (2022) 28(4):658–65. doi: 10.1038/s41591-022-01717-2 35440717

[120] Sheftel AD Stehling O Pierik AJ Elsässer HP Mühlenhoff U Webert H. Humans possess two mitochondrial ferredoxins, Fdx1 and Fdx2, with distinct roles in steroidogenesis, heme, and Fe/S cluster biosynthesis. Proc Natl Acad Sci United States America (2010) 107(26):11775–80. doi: 10.1073/pnas.1004250107 PMC2900682 20547883

[121] Yang L Zhang Y Wang Y Jiang P Liu F Feng N. Ferredoxin 1 is a cuproptosis-key gene responsible for tumor immunity and drug sensitivity: a pan-cancer analysis. Front Pharmacol (2022) 13:938134. doi: 10.3389/fphar.2022.938134 36210836 PMC9532935

[122] Wang T Liu Y Li Q Luo Y Liu D Li B. Cuproptosis-related gene FDX1 expression correlates with the prognosis and tumor immune microenvironment in clear cell renal cell carcinoma. Front Immunol (2022) 13:999823. doi: 10.3389/fimmu.2022.999823 36225932 PMC9549781

[123] Zhang C Zeng Y Guo X Shen H Zhang J Wang K. Pan-cancer analyses confirmed the cuproptosis-related gene FDX1 as an immunotherapy predictor and prognostic biomarker. Front Genet (2022) 13:923737. doi: 10.3389/fgene.2022.923737 35991547 PMC9388757

[124] Zhang Z Ma Y Guo X Du Y Zhu Q Wang X. FDX1 can impact the prognosis and mediate the metabolism of lung adenocarcinoma. Front Pharmacol (2021) 12:749134. doi: 10.3389/fphar.2021.749134 34690780 PMC8531531

[125] Zhang Z Zeng X Wu Y Liu Y Zhang X Song Z. Cuproptosis-related risk score predicts prognosis and characterizes the tumor microenvironment in hepatocellular carcinoma. Front Immunol (2022) 13:925618. doi: 10.3389/fimmu.2022.925618 35898502 PMC9311491

[126] Hatzistergos KE Williams AR Dykxhoorn D Bellio MA Yu W Hare JM. Tumor suppressors RB1 and CDKN2a cooperatively regulate cell-cycle progression and differentiation during cardiomyocyte development and repair. Circ Res (2019) 124(8):1184–97. doi: 10.1161/circresaha.118.314063 30744497

[127] López-Otín C Blasco MA Partridge L Serrano M Kroemer G. The hallmarks of aging. Cell (2013) 153(6):1194–217. doi: 10.1016/j.cell.2013.05.039 PMC3836174 23746838

[128] Tchkonia T Zhu Y van Deursen J Campisi J Kirkland JL. Cellular senescence and the senescent secretory phenotype: therapeutic opportunities. J Clin Invest (2013) 123(3):966–72. doi: 10.1172/jci64098 PMC3582125 23454759

[129] Manolagas SC. The quest for osteoporosis mechanisms and rational therapies: how far we’ve come, how much further we need to go. J Bone miner Res (2018) 33(3):371–85. doi: 10.1002/jbmr.3400 PMC6816306 29405383

[130] Farr JN Fraser DG Wang H Jaehn K Ogrodnik MB Weivoda MM. Identification of senescent cells in the bone microenvironment. J Bone miner Res (2016) 31(11):1920–29. doi: 10.1002/jbmr.2892 PMC5289710 27341653

[131] Khosla S Farr JN Kirkland JL. Inhibiting cellular senescence: a new therapeutic paradigm for age-related osteoporosis. J Clin Endocrinol Metab (2018) 103(4):1282–90. doi: 10.1210/jc.2017-02694 PMC6276719 29425296

[132] Childs BG Gluscevic M Baker DJ Laberge RM Marquess D Dananberg J. Senescent cells: an emerging target for diseases of ageing. Nat Rev Drug Discovery (2017) 16(10):718–35. doi: 10.1038/nrd.2017.116 PMC5942225 28729727

[133] He S Sharpless NE. Senescence in health and disease. Cell (2017) 169(6):1000–11. doi: 10.1016/j.cell.2017.05.015 PMC5643029 28575665

[134] Riggs BL Khosla S Melton LJ 3rd. Sex steroids and the construction and conservation of the adult skeleton. Endocr Rev (2002) 23(3):279–302. doi: 10.1210/edrv.23.3.0465 12050121

[135] Krishnamurthy J Torrice C Ramsey MR Kovalev GI Al-Regaiey K Su L. Ink4a/Arf expression is a biomarker of aging. J Clin Invest (2004) 114(9):1299–307. doi: 10.1172/jci22475 PMC524230 15520862

[136] Sims NA. Senescent osteocytes: do they cause damage and can they be targeted to preserve the skeleton? J Bone miner Res (2016) 31(11):1917–19. doi: 10.1002/jbmr.2994 27653182

[137] Khosla S Farr JN Tchkonia T Kirkland JL. The role of cellular senescence in ageing and endocrine disease. Nat Rev Endocrinol (2020) 16(5):263–75. doi: 10.1038/s41574-020-0335-y PMC7227781 32161396

[138] Farr JN Xu M Weivoda MM Monroe DG Fraser DG Onken JL. Targeting cellular senescence prevents age-related bone loss in mice. Nat Med (2017) 23(9):1072–79. doi: 10.1038/nm.4385 PMC5657592 28825716

[139] Wouters K Deleye Y Hannou SA Vanhoutte J Maréchal X Coisne A. The tumour suppressor CDKN2A/p16(INK4a) regulates adipogenesis and bone marrow-dependent development of perivascular adipose tissue. Diabetes Vasc Dis Res (2017) 14(6):516–24. doi: 10.1177/1479164117728012 PMC5652646 28868898

[140] Kim HN Chang J Iyer S Han L Campisi J Manolagas SC. Elimination of senescent osteoclast progenitors has no effect on the age-associated loss of bone mass in mice. Aging Cell (2019) 18(3):e12923. doi: 10.1111/acel.12923 30773784 PMC6516158

[141] Yang R Chen J Zhang J Qin R Wang R Qiu Y. 1,25-dihydroxyvitamin d protects against age-related osteoporosis by a novel VDR-Ezh2-p16 signal axis. Aging Cell (2020) 19(2):e13095. doi: 10.1111/acel.13095 31880094 PMC6996957

[142] Chen G Zhang Y Yu S Sun W Miao D. Bmi1 overexpression in mesenchymal stem cells exerts antiaging and antiosteoporosis effects by inactivating p16/p19 signaling and inhibiting oxidative stress. Stem Cells (Dayton Ohio) (2019) 37(9):1200–11. doi: 10.1002/stem.3007 PMC6851636 30895687

[143] Kua HY Liu H Leong WF Li L Jia D Ma G. C-abl promotes osteoblast expansion by differentially regulating canonical and non-canonical BMP pathways and p16INK4a expression. Nat Cell Biol (2012) 14(7):727–37. doi: 10.1038/ncb2528 PMC8265173 22729085

[144] Lee SY Park KH Lee G Kim SJ Song WH Kwon SH. Hypoxia-inducible factor-2α mediates senescence-associated intrinsic mechanisms of age-related bone loss. Exp Mol Med (2021) 53(4):591–604. doi: 10.1038/s12276-021-00594-y 33811248 PMC8102580

[145] Cudejko C Wouters K Fuentes L Hannou SA Paquet C Bantubungi K. p16INK4a deficiency promotes IL-4-induced polarization and inhibits proinflammatory signaling in macrophages. Blood (2011) 118(9):2556–66. doi: 10.1182/blood-2010-10-313106 PMC3677739 21636855

[146] Moran JF Sun Z Sarath G Arredondo-Peter R James EK Becana M. Molecular cloning, functional characterization, and subcellular localization of soybean nodule dihydrolipoamide reductase. Plant Physiol (2002) 128(1):300–13. doi: 10.1104/pp.010505 PMC149001 11788775

[147] Ciszak EM Makal A Hong YS Vettaikkorumakankauv AK Korotchkina LG Patel MS. How dihydrolipoamide dehydrogenase-binding protein binds dihydrolipoamide dehydrogenase in the human pyruvate dehydrogenase complex. J Biol Chem (2006) 281(1):648–55. doi: 10.1074/jbc.M507850200 16263718

[148] Danson MJ Conroy K McQuattie A Stevenson KJ. Dihydrolipoamide dehydrogenase from trypanosoma brucei. characterization and cellular location. Biochem J (1987) 243(3):661–5. doi: 10.1042/bj2430661 PMC1147910 3663096

[149] Chiranjivi AK Dubey VK. Dihydrolipoamide dehydrogenase from leishmania donovani: new insights through biochemical characterization. Int J Biol macromol (2018) 112:1241–47. doi: 10.1016/j.ijbiomac.2018.02.112 29466712

[150] Xi Y Pan W Liu Y Liu J Xu G Su Y. α-lipoic acid loaded hollow gold nanoparticles designed for osteoporosis treatment: preparation, characterization and in vitro evaluation. Artif cells nanomed Biotechnol (2023) 51(1):131–38. doi: 10.1080/21691401.2022.2149542 36912372

[151] Dörsam B Fahrer J. The disulfide compound α-lipoic acid and its derivatives: a novel class of anticancer agents targeting mitochondria. Cancer Lett (2016) 371(1):12–9. doi: 10.1016/j.canlet.2015.11.019 26604131

[152] Karner CM Long F. Glucose metabolism in bone. Bone (2018) 115:2–7. doi: 10.1016/j.bone.2017.08.008 28843700 PMC6030501

[153] Ahmad W. Dihydrolipoamide dehydrogenase suppression induces human tau phosphorylation by increasing whole body glucose levels in a c. elegans model of alzheimer’s disease. Exp Brain Res (2018) 236(11):2857–66. doi: 10.1007/s00221-018-5341-0 30056470

[154] Cipriani C Colangelo L Santori R Renella M Mastrantonio M Minisola S. The interplay between bone and glucose metabolism. Front Endocrinol (2020) 11:122. doi: 10.3389/fendo.2020.00122 PMC7105593 32265831

[155] Lee JM Kim MJ Lee SJ Kim BG Choi JY Lee SM. PDK2 deficiency prevents ovariectomy-induced bone loss in mice by regulating the RANKL-NFATc1 pathway during osteoclastogenesis. J Bone miner (2021) 36(3):553–66. doi: 10.1002/jbmr.4202 33125772

[156] Fleminger G Dayan A. The moonlighting activities of dihydrolipoamide dehydrogenase: biotechnological and biomedical applications. J Mol recognit: JMR (2021) 34(11):e2924. doi: 10.1002/jmr.2924 34164859

[157] Dayan A Lamed R Benayahu D Fleminger G. RGD-modified dihydrolipoamide dehydrogenase as a molecular bridge for enhancing the adhesion of bone forming cells to titanium dioxide implant surfaces. J Biomed materials Res Part A (2019) 107(3):545–51. doi: 10.1002/jbm.a.36570 30390369

[158] Puleo DA Bizios R. RGDS tetrapeptide binds to osteoblasts and inhibits fibronectin-mediated adhesion. Bone (1991) 12(4):271–6. doi: 10.1016/8756-3282(91)90075-t 1793678

[159] Radtke F Heuchel R Georgiev O Hergersberg M Gariglio M Dembic Z. Cloned transcription factor MTF-1 activates the mouse metallothionein I promoter. EMBO J (1993) 12(4):1355–62. doi: 10.1002/j.1460-2075.1993.tb05780.x PMC413347 8467794

[160] Zhang D Zhang T Liu J Chen J Li Y Ning G. Zn supplement-antagonized cadmium-induced cytotoxicity in macrophages in vitro: involvement of cadmium bioaccumulation and metallothioneins regulation. J Agric Food Chem (2019) 67(16):4611–22. doi: 10.1021/acs.jafc.9b00232 30942077

[161] Rutherford JC Bird AJ. Metal-responsive transcription factors that regulate iron, zinc, and copper homeostasis in eukaryotic cells. Eukaryotic Cell (2004) 3(1):1–13. doi: 10.1128/ec.3.1.1-13.2004 14871932 PMC329510

[162] Andrews GK. Cellular zinc sensors: MTF-1 regulation of gene expression. Biometals (2001) 14(3-4):223–37. doi: 10.1023/a:1012932712483 11831458

[163] Tavera-Montañez C Hainer SJ Cangussu D Gordon SJV Xiao Y Reyes-Gutierrez P. The classic metal-sensing transcription factor MTF1 promotes myogenesis in response to copper. FASEB J (2019) 33(12):14556–74. doi: 10.1096/fj.201901606R PMC6894080 31690123

[164] Hübner C Haase H. Interactions of zinc- and redox-signaling pathways. Redox Biol (2021) 41:101916. doi: 10.1016/j.redox.2021.101916 33662875 PMC7937829

[165] Wong SH Zhao Y Schoene NW Han CT Shih RS Lei KY. Zinc deficiency depresses p21 gene expression: inhibition of cell cycle progression is independent of the decrease in p21 protein level in HepG2 cells. Am J Physiol Cell Physiol (2007) 292(6):C2175–84. doi: 10.1152/ajpcell.00256.2006 17303651

[166] John E Laskow TC Buchser WJ Pitt BR Basse PH Butterfield LH. Zinc in innate and adaptive tumor immunity. J Trans Med (2010) 8:118. doi: 10.1186/1479-5876-8-118 PMC3002329 21087493

[167] Cousins RJ Aydemir TB Lichten LA. Plenary lecture 2: transcription factors, regulatory elements and nutrient-gene communication. Proc Nutr Soc (2010) 69(1):91–4. doi: 10.1017/s0029665109991790 PMC3790273 19968906

[168] Liuzzi JP Wong CP Ho E Tracey A. Regulation of hepatic suppressor of cytokine signaling 3 by zinc. J Nutr Biochem (2013) 24(6):1028–33. doi: 10.1016/j.jnutbio.2012.07.011 23026491

[169] Lichtlen P Schaffner W. The “metal transcription factor” MTF-1: biological facts and medical implications. Swiss Med weekly (2001) 131(45-46):647–52. doi: 10.4414/smw.2001.09672 11835113

[170] Beaver LM Nkrumah-Elie YM Truong L Barton CL Knecht AL Gonnerman GD. Adverse effects of parental zinc deficiency on metal homeostasis and embryonic development in a zebrafish model. J Nutr Biochem (2017) 43:78–87. doi: 10.1016/j.jnutbio.2017.02.006 28268202 PMC5406264

[171] Dong G Chen H Qi M Dou Y Wang Q. Balance between metallothionein and metal response element binding transcription factor 1 is mediated by zinc ions (review). Mol Med Rep (2015) 11(3):1582–6. doi: 10.3892/mmr.2014.2969 25405524

[172] Cannino G Ferruggia E Luparello C Rinaldi AM. Cadmium and mitochondria. Mitochondrion (2009) 9(6):377–84. doi: 10.1016/j.mito.2009.08.009 19706341

[173] Park JD Liu Y Klaassen CD. Protective effect of metallothionein against the toxicity of cadmium and other metals(1). Toxicology (2001) 163(2-3):93–100. doi: 10.1016/s0300-483x(01)00375-4 11516518

[174] Miles AT Hawksworth GM Beattie JH Rodilla V. Induction, regulation, degradation, and biological significance of mammalian metallothioneins. Crit Rev Biochem Mol Biol (2000) 35(1):35–70. doi: 10.1080/10409230091169168 10755665

[175] Smith A. Links between cell-surface events involving redox-active copper and gene regulation in the hemopexin heme transport system. Antioxid Redox Signaling (2000) 2(2):157–75. doi: 10.1089/ars.2000.2.2-157 11229523

[176] Saydam N Georgiev O Nakano MY Greber UF Schaffner W. Nucleo-cytoplasmic trafficking of metal-regulatory transcription factor 1 is regulated by diverse stress signals. J Biol Chem (2001) 276(27):25487–95. doi: 10.1074/jbc.M009154200 11306562

[177] Labbé S Prévost J Remondelli P Leone A Séguin C. A nuclear factor binds to the metal regulatory elements of the mouse gene encoding metallothionein-I. Nucleic Acids Res (1991) 19(15):4225–31. doi: 10.1093/nar/19.15.4225 PMC328566 1870976

[178] Larochelle O Stewart G Moffatt P Tremblay V Séguin C. Characterization of the mouse metal-regulatory-element-binding proteins, metal element protein-1 and metal regulatory transcription factor-1. Biochem J (2001) 353(Pt 3):591–601. doi: 10.1042/0264-6021:3530591 11171056 PMC1221605

[179] Chen PH Wu J Ding CC Lin CC Pan S Bossa N. Kinome screen of ferroptosis reveals a novel role of ATM in regulating iron metabolism. Cell Death differ (2020) 27(3):1008–22. doi: 10.1038/s41418-019-0393-7 PMC7206124 31320750

[180] Lyu Z Yang M Yang T Ma M Yang Z. Metal-regulatory transcription factor-1 targeted by miR-148a-3p is implicated in human hepatocellular carcinoma progression. Front Oncol (2021) 11:700649. doi: 10.3389/fonc.2021.700649 34660270 PMC8511627

[181] Li Y Zeng X. A novel cuproptosis-related prognostic gene signature and validation of differential expression in hepatocellular carcinoma. Front Pharmacol (2022) 13:1081952. doi: 10.3389/fphar.2022.1081952 36703728 PMC9871247

[182] Tort F Ferrer-Cortès X Thió M Navarro-Sastre A Matalonga L Quintana E. Mutations in the lipoyltransferase LIPT1 gene cause a fatal disease associated with a specific lipoylation defect of the 2-ketoacid dehydrogenase complexes. Hum Mol Genet (2014) 23(7):1907–15. doi: 10.1093/hmg/ddt585 24256811

[183] Fan X Chen H Jiang F Xu C Wang Y Wang H. Comprehensive analysis of cuproptosis-related genes in immune infiltration in ischemic stroke. Front Neurol (2022) 13:1077178. doi: 10.3389/fneur.2022.1077178 36818726 PMC9933552

[184] Lv H Liu X Zeng X Liu Y Zhang C Zhang Q. Comprehensive analysis of cuproptosis-related genes in immune infiltration and prognosis in melanoma. Front Pharmacol (2022) 13:930041. doi: 10.3389/fphar.2022.930041 35837286 PMC9273972

[185] Solmonson A Faubert B Gu W Rao A Cowdin MA Menendez-Montes I. Compartmentalized metabolism supports midgestation mammalian development. Nature (2022) 604(7905):349–53. doi: 10.1038/s41586-022-04557-9 PMC9007737 35388219

[186] Ni M Solmonson A Pan C Yang C Li D Notzon A. Functional assessment of lipoyltransferase-1 deficiency in cells, mice, and humans. Cell Rep (2019) 27(5):1376–86.e6. doi: 10.1016/j.celrep.2019.04.005 31042466 PMC7351313

[187] Wang X Xie C Lin L. Development and validation of a cuproptosis-related lncRNA model correlated to the cancer-associated fibroblasts enable the prediction prognosis of patients with osteosarcoma. J Bone Oncol (2023) 38:100463. doi: 10.1016/j.jbo.2022.100463 36569351 PMC9772846

[188] Liu B Liu Z Feng C Li C Zhang H Li Z. Identification of cuproptosis-related lncRNA prognostic signature for osteosarcoma. Front Endocrinol (2022) 13:987942. doi: 10.3389/fendo.2022.987942 PMC9606239 36313774

[189] Yang M Zheng H Xu K Yuan Q Aihaiti Y Cai Y. A novel signature to guide osteosarcoma prognosis and immune microenvironment: cuproptosis-related lncRNA. Front Immunol (2022) 13:919231. doi: 10.3389/fimmu.2022.919231 35967366 PMC9373797

[190] Yang W Wu H Tong L Wang Y Guo Q Xu L. A cuproptosis-related genes signature associated with prognosis and immune cell infiltration in osteosarcoma. Front Oncol (2022) 12:1015094. doi: 10.3389/fonc.2022.1015094 36276092 PMC9582135

[191] Jiang J Zhan X Wei J Fan Q Li H Li H. Artificial intelligence reveals dysregulation of osteosarcoma and cuproptosis-related biomarkers, PDHA1, CDKN2A and neutrophils. Sci Rep (2023) 13(1):4927. doi: 10.1038/s41598-023-32195-2 36967449 PMC10040405

[192] Su Y Xu C Sun Z Liang Y Li G Tong T. S100A13 promotes senescence-associated secretory phenotype and cellular senescence via modulation of non-classical secretion of IL-1α. Aging (2019) 11(2):549–72. doi: 10.18632/aging.101760 PMC6366962 30670674

[193] Masaldan S Clatworthy SAS Gamell C Smith ZM Francis PS Denoyer D. Copper accumulation in senescent cells: interplay between copper transporters and impaired autophagy. Redox Biol (2018) 16:322–31. doi: 10.1016/j.redox.2018.03.007 PMC5953000 29579719

[194] Matos L Gouveia AM Almeida H. ER stress response in human cellular models of senescence. journals gerontol Ser A Biol Sci Med Sci (2015) 70(8):924–35. doi: 10.1093/gerona/glu129 25149687

[195] Matos L Gouveia AM Almeida H. Resveratrol attenuates copper-induced senescence by improving cellular proteostasis. Oxid Med Cell Longevity (2017) 2017:3793817. doi: 10.1155/2017/3793817 PMC5322428 28280523

